# Transcriptome profiling of cells exposed to particular and intense electromagnetic radiation emitted by the "SG-III" prototype laser facility

**DOI:** 10.1038/s41598-021-81642-5

**Published:** 2021-01-21

**Authors:** Jiangbin Wei, Qiwu Shi, Lidan Xiong, Guang Xin, Tao Yi, Yunqing Xiao, Wanxia Huang

**Affiliations:** 1 College of Materials Science and Engineering, Sichuan University, Chengdu, 610065 Sichuan China; 2 Department of Dermatology, West China Hospital, Sichuan University, Chengdu, 610041 China; 3 Laboratory of Ethnopharmacology, West China School of Medicine, West China Hospital, Sichuan University, Chengdu, 610041 Sichuan China; 4 Research Center of Laser Fusion, China Academy of Engineering Physics, Mianyang, 621900 China

**Keywords:** Biophysics, Cell biology, Genetics, Molecular biology, Neuroscience

## Abstract

The experiment of inertial confinement fusion by the “ShengGuang (SG)-III” prototype laser facility is a transient and extreme reaction process within several nanoseconds, which could form a very complicated and intense electromagnetic field around the target chamber of the facility and may lead to harmful effect on people around. In particular, the biological effects arising from such specific environment field could hardly be ignored and have never been investigated yet, and thus, we reported on the investigation of the biological effects of radiation on HaCat cells and PC12 cells to preliminarily assess the biological safety of the target range of the "SG-III" prototype laser facility. The viability revealed that the damage of cells was dose-dependent. Then we compared the transcriptomes of exposed and unexposed PC12 cells by RNA-Seq analysis based on Illumina Novaseq 6000 platform and found that most significantly differentially expressed genes with corresponding Gene Ontology terms and pathways were strongly involved in proliferation, transformation, necrosis, inflammation response, apoptosis and DNA damage. Furthermore, we find increase in the levels of several proteins responsible for cell-cycle regulation and tumor suppression, suggesting that pathways or mechanisms regarding DNA damage repair was are quickly activated. It was found that "SG-III" prototype radiation could induce DNA damage and promote apoptotic necrosis.

## Introduction

With the steady development in high power solid state laser technologies for application to Inertial confinement fusion (ICF) recent decades, the famous fusion facilities were constructed, including the National Ignition Facility (NIF)^1^, the French Megajoule Laser Project (LMJ)^2^, Japanese Gekko XII and the Chinese “SG” series facilities, which strongly promoted the research of laser fusion. China has independently developed and built several high-power laser facilities from “XinGuang (XG)-I” to “ShengGuang (SG)-III” prototype laser facility. As a second-generation high-power solid-state laser facility, the "SG-III" prototype laser facility, was successfully developed at the Chinese Academy of Engineering Physics at the end of 2007^3^. It has been the first high-power laser facility in China characterized by "square beam, combined aperture, and multi-pass amplification", which had been the first high-power laser facility in Asia and one of the few high-power laser facilities, whose energy of third Harmonic Generation exceeds the degree of ten thousand joules in the world. However, the process of ICF experiments of the "SG-III" laser facility is a transient and extremely drastic reaction with intense electromagnetic pulse radiation, X-ray pulses, high temperature plasma, hot electrons and superheated electrons generated when high-power laser shoots the target^4,5^. The accelerated motion and echo oscillations of high-temperature plasma (including a large number of charged particles) could generate intense electromagnetic pulse radiation, which could form a very complex electromagnetic environment near the target chamber and result in the inevitable and inconvenient biological effects that have never been investigated yet. And the "SG-III" prototype laser facility is the only particular facility for us to perform biological researches and it is hard for visitors to have access to it, So we made every effort to conduct some biological experiments in such specific form of radiation to obtain the precious cell samples.

In this paper, HaCat and PC12 cells, which was described a common keratinocyte and neurocyte model respectively, were used as the vitro toxicological research models and the cell samples were placed around the chamber at different locations. The electromagnetic radiation generated by this facility were used to irradiate the two type of cells during the laser targeting. The irradiation dose for cells was negatively correlated with the distance between the cell samples and the center of the target chamber, and the electromagnetic radiation energy at the window of the target chamber was greatest, because electromagnetic pulses, X-ray pulses, and other particles were more likely to pass through the chamber wall. The cytotoxicity experiment and RNA-Seq were performed to preliminarily evaluate the compound effect in terms of cells and genes, and then preliminarily discussed the mechanism of DNA damage and repair induced by "SG-III" prototype radiation through ELISA, Westernblot and qRT-PCR experiments.

## Results

Samples were collected from PC12 cells and HaCat cells. The cell viability tested by CCK-8 method through the cytotoxicology experiments and then RNA-Seq were performed to initially evaluate the biological safety effect of the "SG-III" prototype laser facility. We conducted the transcriptome profiling analysis of the differentially expressed genes (DEGs) of PC12 cells and corresponding Gene Ontology functional enrichment analysis, and KEGG pathways enrichment analysis were carried out by using clusterProfiler software for the differential gene set to reveal some biological effects.

### Cytotoxicological analysis based on ICF experiment of "SG -III" prototype laser facility

**Figure 1:**
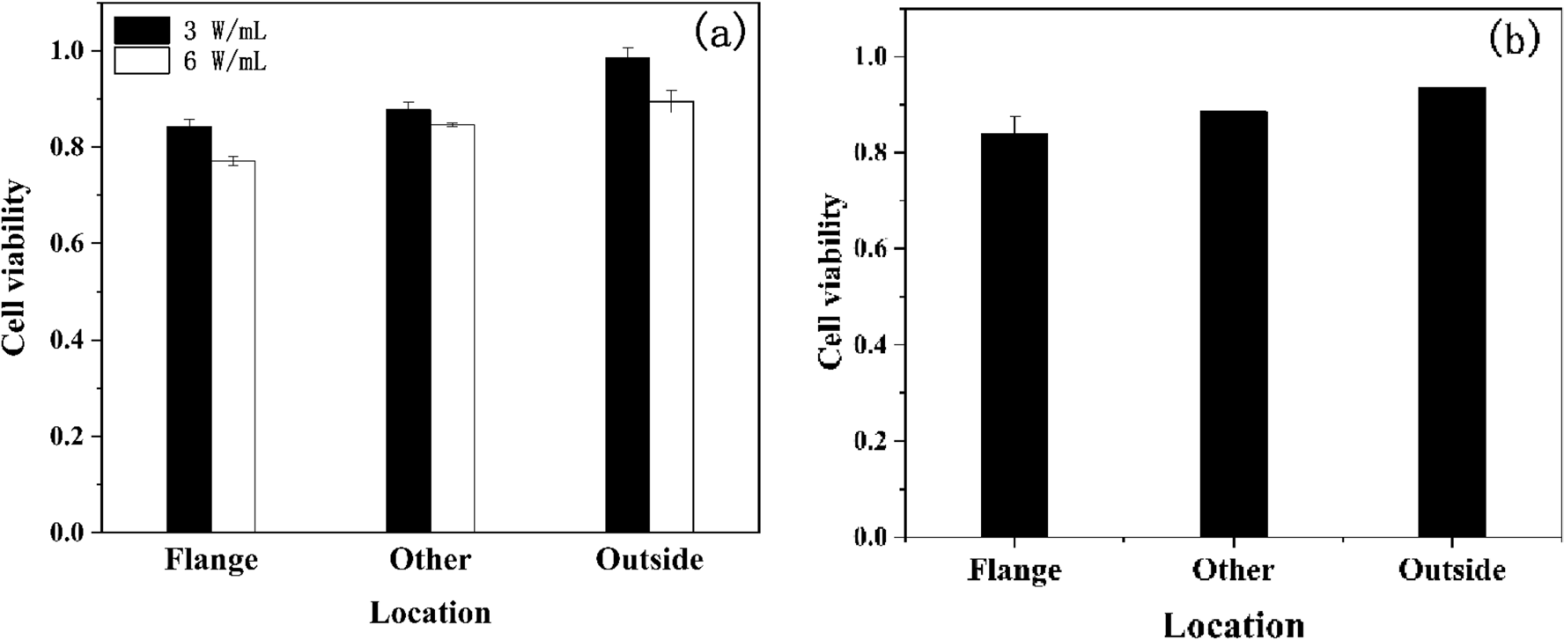
Viability of PC12 cells with a concentration of 3 W/mL and 6 W/mL (**a**) and HaCat cells With a concentration of 6 W/mL (**b**) irradiated by the the "SG-III" prototype laser facility at different locations of the target range (“Flange” represents location near the window of the chamber, “Other” represents location away from the chamber, “Outside” represents location outside the target range, so the radiation dose increased sequentially from Flange to Outside).

The effect of ICF experiment of the "SG-III" prototype laser facility on the viability of PC12 cells and HaCat cells with different concentrations (Fig. 1).

As shown in 1,444,444. 1, the damage degree of two kinds of cells is1 positively correlated with the energy of the target of the SG-III prototype facility. The viability of cells in High group was about 15 percentage points lower than in Low group. In addition, we could detect intense radiation energy in the site of High group, which means electromagnetic pulse radiation, X-ray pulse, high temperature plasma, hot electrons and superheated electrons are more likely to transmit through the target chamber wall and aggregate around the chamber.

### Analysis of mapping rates between reads and reference genomes

To investigate the transcriptome response to cells exposed to irradiation from the the "SG-III" prototype laser facility and attempt to know about potential effect and risk to staff, we performed RNA-Seq to measure mRNA transcriptome profiles in Control, Low, and High Group. Illumina Sequencing platform was used to perform high-throughput RNA-Seq analysis on cells libraries that were constructed at radiation treatment and control. Prior to mapping such sequences to the reference sequences, reads with adaptor sequences, reads with N sequences (N means that the base information cannot be determined), and reads with low quality sequences (reads with Qphred ≤ 20 base number accounting for more than 50% of the total read length) were filtered to ensure the quality and reliability of data analysis, producing approximately 46 million total reads (clean reads) per sample and with about 42 million total mapping reads (number of reads mapping to the reference genome). We could see that the percentage of clean reads among raw reads of Single-read each group in each library ranged from 90.16 to 95.36% (Supplementary Fig. S1) and the mapping rates of total reads (clean reads) to the reference mouse genome in all samples were above 92% (Table 1), indicating that the species had a high homology with the chosed reference specie. The mapping rate between the samples and the reference genome is as follows. The number of total_reads in the following table is the sum of read1 and read2, that is clean reads aboved.

**Table 1:** Statistical tables of mapped reads of paired-end.

Sample_ID	total_reads	total_map	unique_map	multi_map	read1_map	read2_map	positive_map	negative_map
Control	45,139,792	42,281,404 (93.67%)	41,312,692 (91.52%)	968,712 (2.15%)	20,714,062 (45.89%)	20,598,630 (45.63%)	20,648,794 (45.74%)	20,663,898 (45.78%)
Low	45,879,270	42,769,425 (93.22%)	41,704,658 (90.9%)	1,064,767 (2.32%)	20,905,769 (45.57%)	20,798,889 (45.33%)	20,843,571 (45.43%)	20,861,087 (45.47%)
High	45,898,106	42,915,108 (93.5%)	41,790,531 (91.05%)	1,124,577 (2.45%)	20,935,227 (45.61%)	20,855,304 (45.44%)	20,887,532 (45.51%)	20,902,999 (45.54%)

total_reads: The number of clean reads sequencing data after quality control.

total_map: The number and percentage of clean reads compared to the reference genome unique_map: The number and percentage of reads at unique locations compared to the reference genome.

multi_map: The number and percentage of reads at multiple locations compared to the reference genome.

read1_map: The number and percentage of read1 compared to the reference genome.

read2_map: The number and percentage of read1 compared to the reference genome.

positive_map: The number and percentage of reads on the positive chain compared to the reference genome.

negative_map: The number and percentage of reads on the negative chain compared to the reference genome.

### Analysis of the correlation between the samples

**Figure 2:**
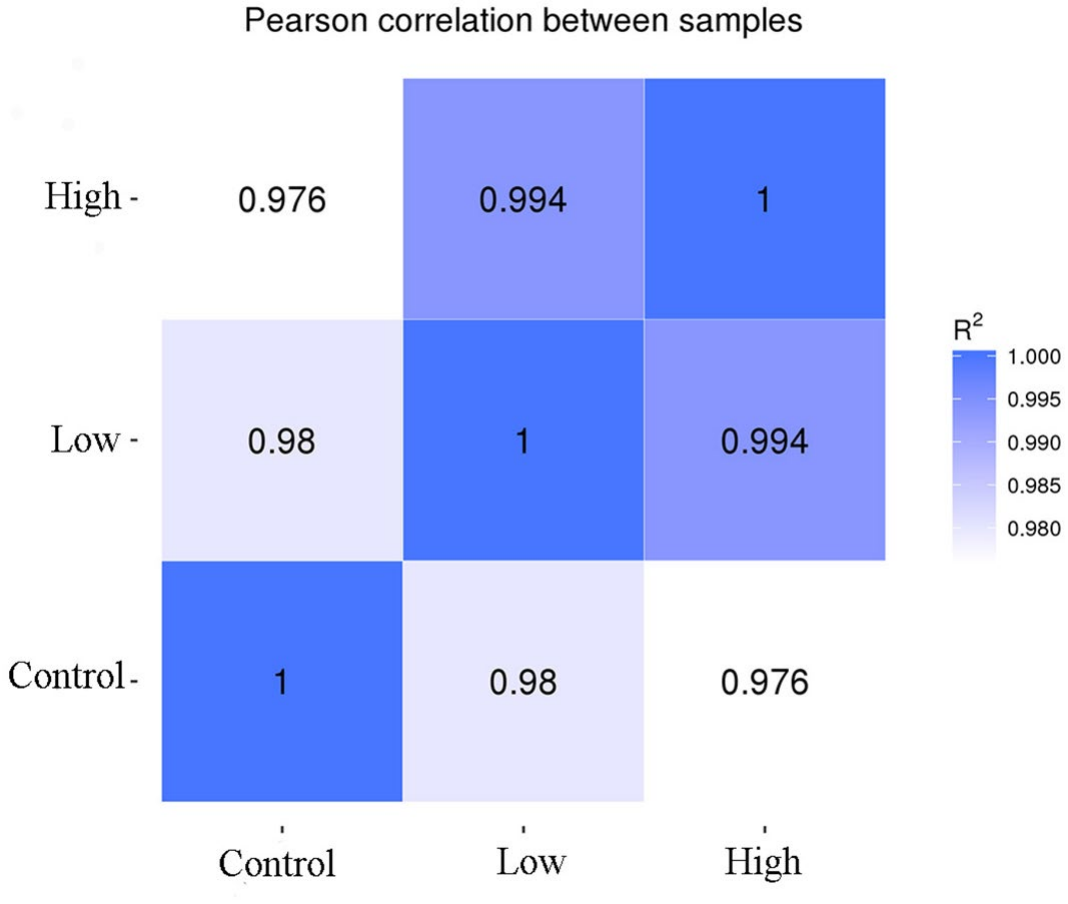
Correlation heatmap between samples (horizontal axis is the square of correlation coefficient of each sample).

The correlation coefficient of samples was calculated, and the heat map was drawn according to the expression value of all genes in each sample (Fig. 2). The results of the correlation heat map showed that the correlation between different samples is more than 97%, which was much greater than 0.8, indicating that the correlation coefficient between samples is very high and the expression patterns are close and also proved that the experimental model is reliable.

**Figure 3:**
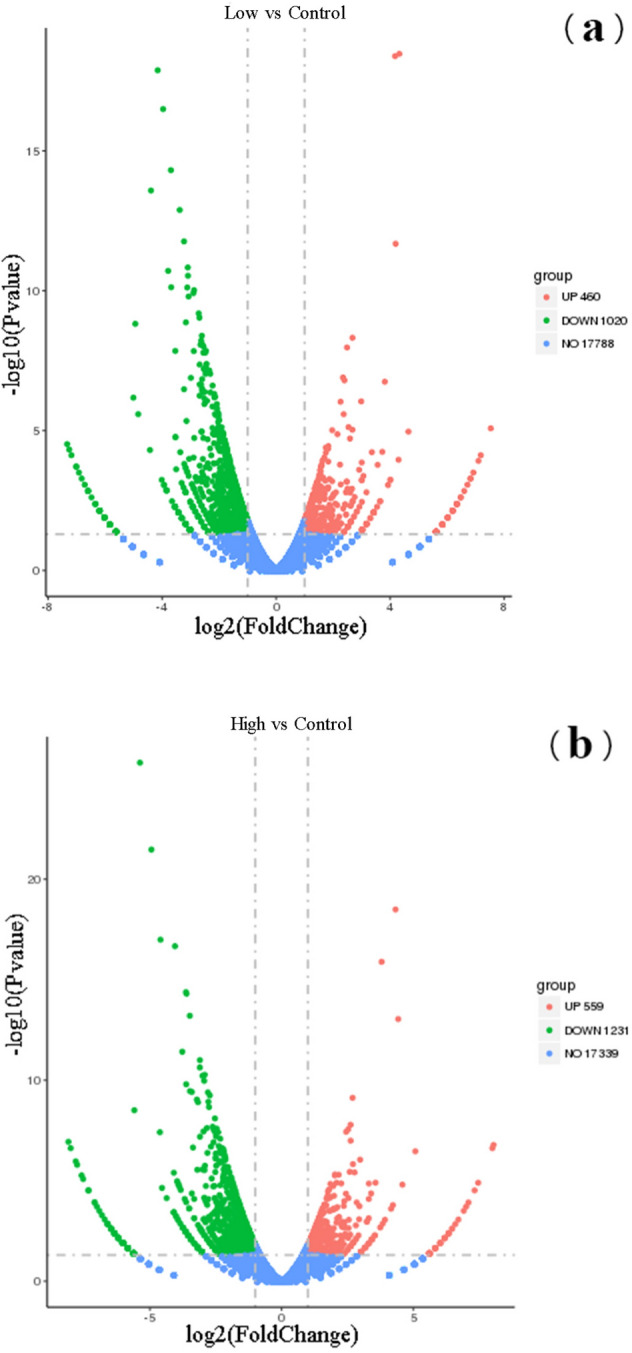
Volcanic map of DEGs between exposed and unexposed PC12 cells in the ICF experiment of "SG- III" prototype laser facility (Abscissa is log2 FoldChange, ordinate is -log10 (*P*-value), blue dotted line represents the threshold line of differential gene screening criteria).

The result of DEGs analysis was shown in Fig. 3. “Control” was the group with no radiation, “Low” was the group outside the target range, and “High” was group inside the target range.

**Figure 4:**
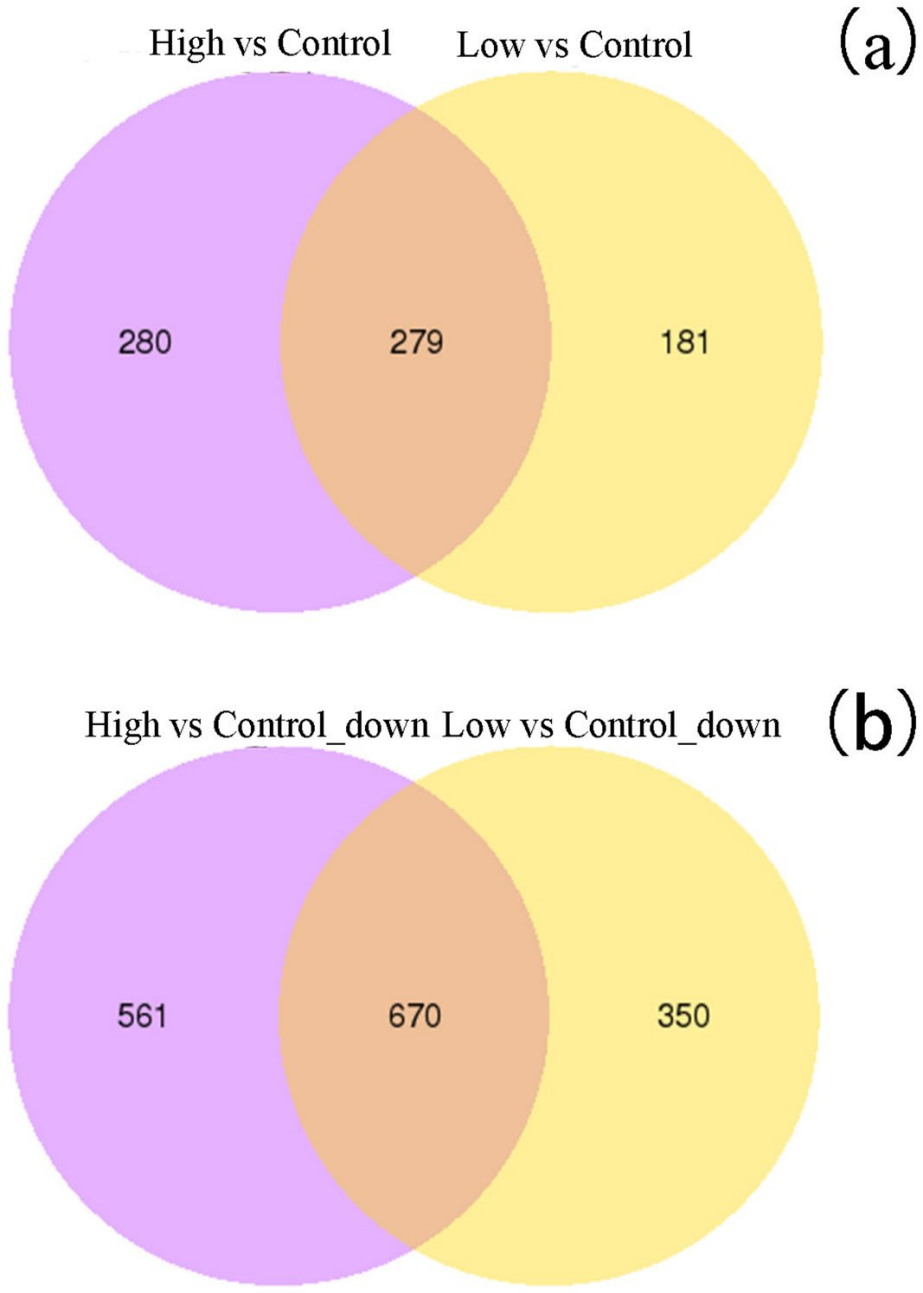
Wayne diagram of DEGs in PC12 cells in the ICF experiment of the "SG-III" prototype laser facility (different colors represent different comparative combinations).

From the volcano map of differential genes,1480 genes were differentially expressed more than 2 times in Low group compared with the Control group, including 460 up-regulated genes and 1020 down-regulated genes, and 1790 genes were more than two-fold differentially expressed in the High group compared with the Control group, including 559 up-regulated genes and 1231 down-regulated genes (Fig. 3). We could also find there were 949 DEGs which Low and High group had in common, including 279 up-regulated genes (Fig. 4a) and 670 down-regulated genes (Fig. 4b). In particular, 84 dose-dependent genes filtrated from DEGs might play an important role in biological and physiological processes, including 20 positively related genes and 64 negatively related genes among the three groups, and the most significantly enriched 20 DEGs were listed respectively (Supplementary Table S1 and Supplementary Table S2).

**Figure 5:**
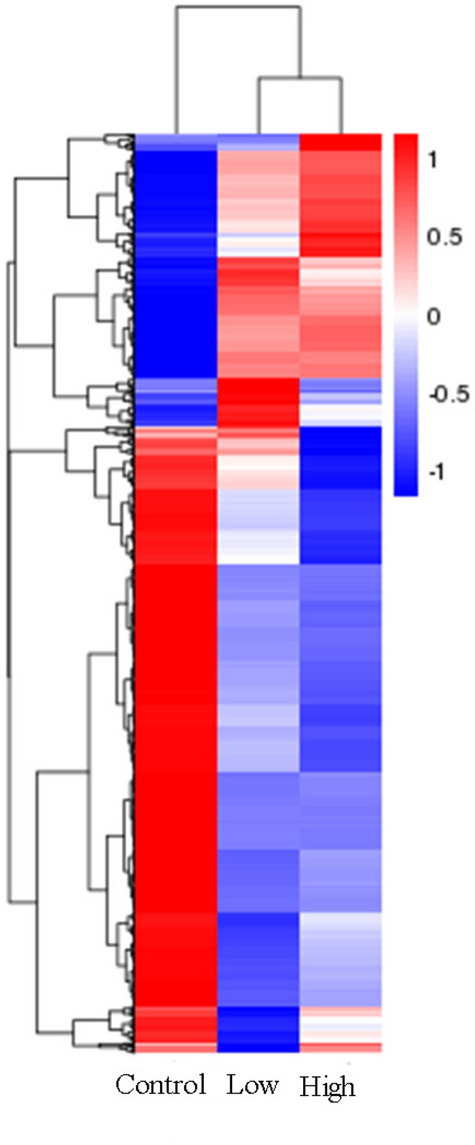
The clustering heat map of DEGs. The Abscissa is the sample name, and the ordinate is the normalized value of the differential gene FPKM. (The redder the color is, the higher the expression is, and the greener the expression is, the lower the expression is).

The samples of each group were analyzed by hierarchical clustering method, and the results (Fig. 5) revealed that Low and High group had the highest similarity of gene expression profiles, then followed by the Control group. At the same time, it can also be found that the expression of gene in the experimental groups (Low and High group) were evidently different from that in the Control group, but the gene expression in the experimental group showed a similar tendency, which mainly existed some difference in the amount of gene expression.

### Analysis of the gene ontology (GO) functional enrichment of DEGs on PC12 cells

Genes with altered expression responses spanned wide varieties of regulatory and metabolic processes. The DEGs of each sample reads were classified into several categories based on their GO terms using clusterProfiler software to analyze the functional enrichment. GO is a comprehensive database to describe the gene function, which can be divided into three main parts: biological process, cellular component and molecular function^6^. The basic unit of GO is regarded as GO term and each GO term belongs to a sort of ontology. GO enrichment analysis provides all GO terms that are significantly enriched in DEGs compared to the genome background. The GO annotation of these DEGs was presented, which showed the number of function-related DEGs (Supplementary Figs. S2, S3, S4), and the proportion of the number of DEGs described by different functions to the total number of DEGs was showed (Supplementary Fig. S4).

In this study, all the 1480 DEGs in the Low group compared with Contrl group could be categorized into 97 functional terms (Supplementary Fig. S2 a, b, c), in which 44, 13 and 40 functional terms most significantly enriched in DEGs in these three main parts of the GO classification were identified, respectively. Among these functional terms, the terms of "steroid metabolic process", "small molecule biosynthetic process", and “lipid biosynthetic process” in the biological process, "distal axon", “terminal bouton” and “extracellular matrix” in the cellular component, "transcription factor activity, RNA polymerase II proximal promoter sequence-specific DNA binding", “signaling receptor activity” and "molecular transducer activity" in the molecular functions were dominant. 1790 DEGs in the High group compared with Contrl group could be categorized into 106 functional terms (Supplementary Fig. S2 d–f), in which 45, 15 and 46 functional terms most significantly enriched in DEGs in these three main parts of the GO classification were identified, respectively. Among these functional terms, the terms of "lipid biosynthetic process", “small molecule biosynthetic process”, “blood vessel development” and “inflammatory response” in the biological process, “distal axon” “axon part” “presynapse” and “transcription factor complex” in the cellular component, “signaling receptor activity”, "molecular transducer activity" and “transmembrane signaling receptor activity” in the molecular functions were dominant. We could also found that DEGs related to “response to fibroblast growth factor”, “inflammatory response”, “cell proliferation”, “ regulation of neuron apoptotic process”, “regulation of neuron death” transpoter recepter activity and some protein binding were also significantly enriched and most up-regulated and down-regulated DEGs involved in these terms are listed (Table 2), indicating intracellular genes could interact with each other to play essential roles in some biological functions.

**Table 2:** Most up-regulated and down-regulated DEGs involved in important GO terms.

Go term	Up-regulated degs	Down-regulated degs
Response to fibroblast growth factor	Thbs1, Ngfr, Snai2, Ctgf	Egr3, Ccl2, Dusp6, Vegfa, Spry2, Nr4a1
Inflammatory response	Il1rl1, Thbs1, Serpinf1, Wfdc1, Ptafr, Ngfr, Metrnl, Ngf, P2rx1, Nppb, Fcgr2a	Nfkbiz, Ccl2, F3, Ccl7, Itga2, Bmp2, Casp4, Ntrk2, MvkC, xcl1, S1pr3, Cxcl11, Rora, Pla2g5
Regulation of epithelial cell proliferation	Thbs1, Serpinf1, Cdh3, Snai2, Wfdc1, Ngfr	Egr3, Ccl2, F3, Nr4a3, Jaml, Mef2c, Bmp2, Vegfa, Hes1, Nr4a1, Vash2
Cytokine-mediated signaling pathway	Il1rl1, Ackr3, Ngfr, Irf7, Numbl	Egr1, Ccl2, F3, Ccl7, Casp4, Cxcl1, Plcb1, Socs1, Cxcl11, Crebrf
Negative regulation of neuron apoptotic process	Crlf1, Ngfr, Ngf	Ccl2, Nr4a3, Mt1, Ntrk2, Mef2c, Vegfa, Park2, Pink1
Negative regulation of neuron death	Serpinf1, Crlf1, Ngfr, Ngf	Ccl2, Nr4a3, Mt1, Ntrk2, Mef2c, Vegfa, Park2, Atf4, Pink1
Molecular transducer activity	Il1rl1, Ackr3, Ptafr, Ngfr, Uts2r, P2rx1, Ephb4, Ephb3, Tlr5, Fcgr2a	Abca1, F3, Sfrp4, Nr4a3, Itga2, Ntrk2, Chrnb2, Nr4a1, Lef1, S1pr3, Gria2, Rora, Gpr82
Transmembrane signaling receptor activity	Il1rl1, Ackr3, Ptafr, Ngfr, Uts2r, P2rx1, Ephb4, Ephb3, Tlr5, Fcgr2a	Abca1, F3, Sfrp4, Itga2, Ntrk2, Chrnb2, S1pr3, Gria2, Gpr82
Growth factor binding	Thbs1, Ngfr, Ctgf	Col2a1, Nkd2, Vegfa, Ntrk2
Core promoter sequence-specific DNA binding	Irf7	Egr1, Fos, Nr4a3, Nfil3, Mef2c, Mitf, Atf4, Rora, Chd2

### KEGG pathway enrichment analysis of PC12 cells

Genes could play roles in certain biological functions by interacting with each other. And KEGG (Kyoto Encyclopedia of Genes and Genomes) is a comprehensive database that integrates genome, chemistry and system function information, so Pathway-based analysis helps to further understand DEGs biological functions. KEGG pathway enrichment analysis could identify significantly enriched signaling transduction pathways or metabolic pathways in DEGs compared with the genome background. The enrichment degree of KEGG is measured by enrichment factors (Rich factor), P-value and the number of genes enriched in this pathway. Abscissa is the enrichment factor, the higher the Rich factor is, the greater the enrichment degree is. The enrichment factor (Rich factor) is that the number of DEGs in the pathway term divided by the number of all annotated genes in the pathway term. The KEGG enrichment analysis of DEGs was shown (Supplementary Fig. S5, S6, and S7) the major pathways significantly enriched in DEGs compared with Control group.

We found that the KEGG terms of "Steroid biosynthesis", “Cytokine-cytokine receptor interaction”, and "PI3K-Akt signaling pathway" were dominant in group B2 (Supplementary Fig. S5a, S6a, S7a). In the High group, the KEGG terms “Cytokine-cytokine receptor interaction”, “PI3K-Akt signaling pathway” and “MAPK signaling pathway” were dominant (Supplementary Fig. S5b, S6b, S7b). “TNF signaling pathway” and “Transcriptional misregulation in cancer” also played important roles in regulation of cell cycle, proliferation and apoptosis. And most up-regulated and down-regulated DEGs involved in these critical terms are listed (Table 3), indicating intracellular genes could interact with each other to play pivotal roles in some biological functions. We could also found some dominant pathways both Low and High group expressed in common, including “Steroid biosynthesis, Cytokine-cytokine receptor interaction, Viral protein interaction with cytokine and cytokine receptor, TNF signaling pathway, PI3K-Akt signaling pathway, MAPK signaling pathway, Transcriptional misregulation in cancer and ECM-receptor interaction”, so some critical pathways and related DEGs were listed in Table 3.

**Table 3:** Important signaling pathways and major DEGs involved in these pathways.

Signaling pathway	Up-regulated degs	Down-regulated degs
TNF signaling pathway	Map3k14	Fos, Ccl2, Junb, Cxcl1, Birc3, Lif, Atf4
PI3K-Akt signaling pathway	Thbs1, Ngfr, Ngf, Col6a3	Col2a1, Col9a2, Itga2, Vegfa, Ntrk2, Ddit4, Nr4a1, Irs1, Atf4
MAPK signaling pathway	Ngfr, Ngf, Flnc	Fos, Dusp6, Mef2c, Vegfa, Ntrk2, Gadd45b, Nr4a1, Dusp4, Atf4
Transcriptional misregulation in cancer	Ngfr	Nfkbiz, Nr4a3, Dusp6, Mef2c, Gadd45b, Mitf, Birc3
cGMP-PKG signaling pathway	Nppb	Mef2c, Prkg1, Irs1, Plcb1, Atf4, Cngb1
Viral protein interaction with cytokine and cytokine receptor	Ackr3	Ccl2, Ccl7, Cxcl1, Cxcl11
Micrornas in cancer	Thbs1	Vegfa, Mir27b, Ddit4, Spry2, Mir7a-1, Irs1, Socs1
Focal adhesion	Thbs1, Flnc, Col6a3	Col2a1, Col9a2, Itga2, Vegfa, Birc3, Pip5k1b

### Analysis of ELISA, Western blot and qRT-PCR experiments

**Figure 6:**
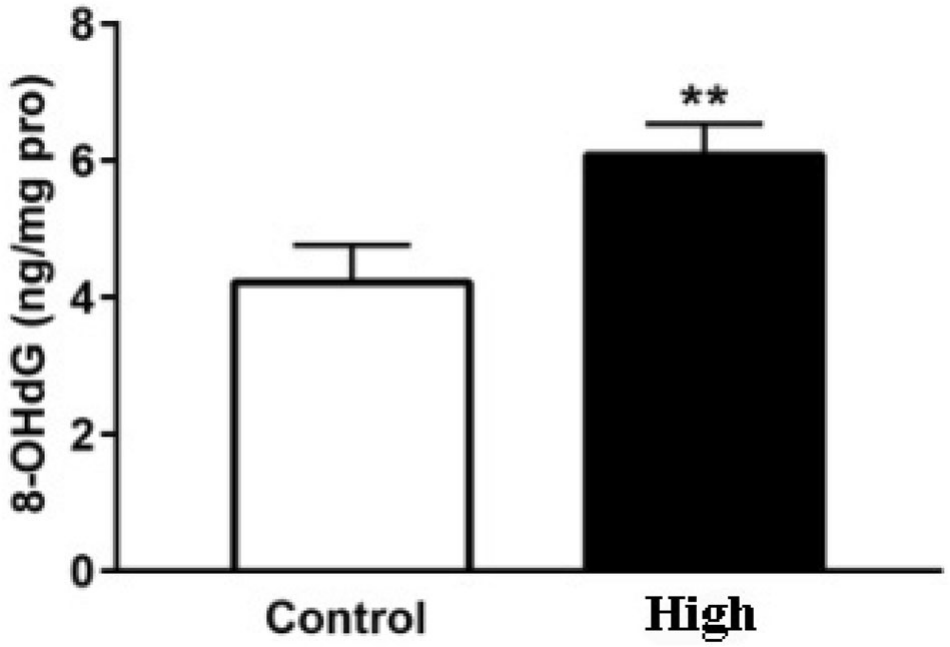
Effects of electromagnetic pulse on 8-OHdG concentration in PC12 cells. The results were presented as mean ± SD (n = 3). **p < 0.01 vs. Control group.

**Figure 7:**
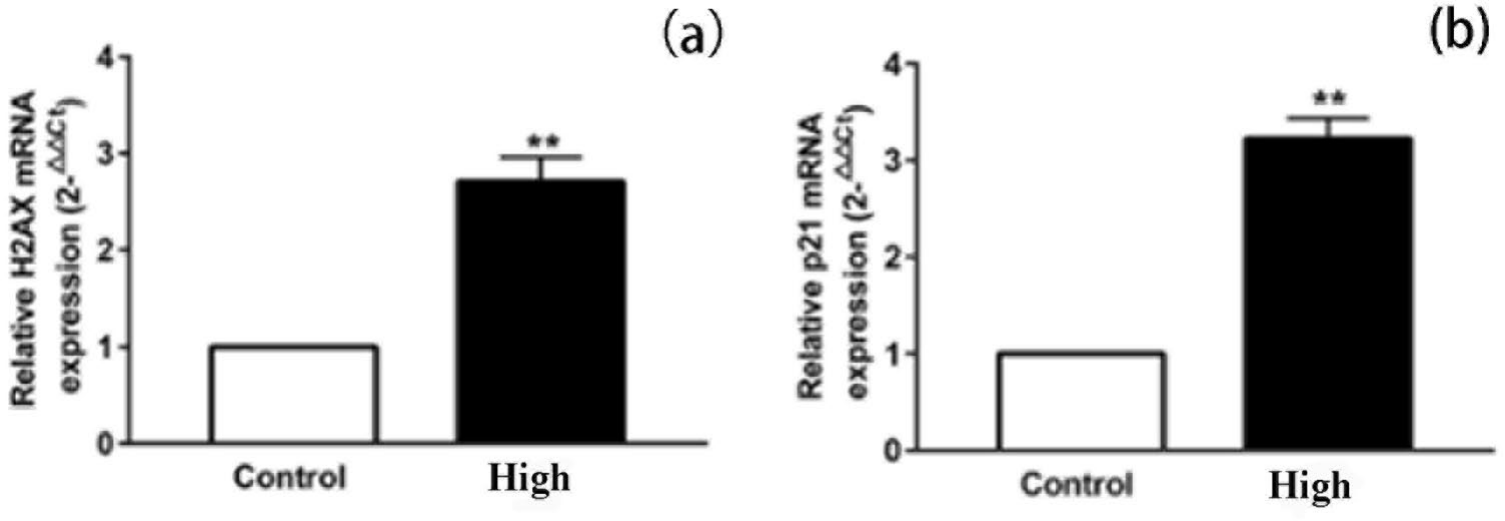
Effect of the mRNA expressions of H2AX and p21 in PC12. The mRNA levels of H2AX and p21were detected by Q-PCR. The mRNA levels of H2AX and p21were normalized to control. The results were presented as mean ± SD (n = 3). **p < 0.01 vs. Control group.

**Figure 8:**
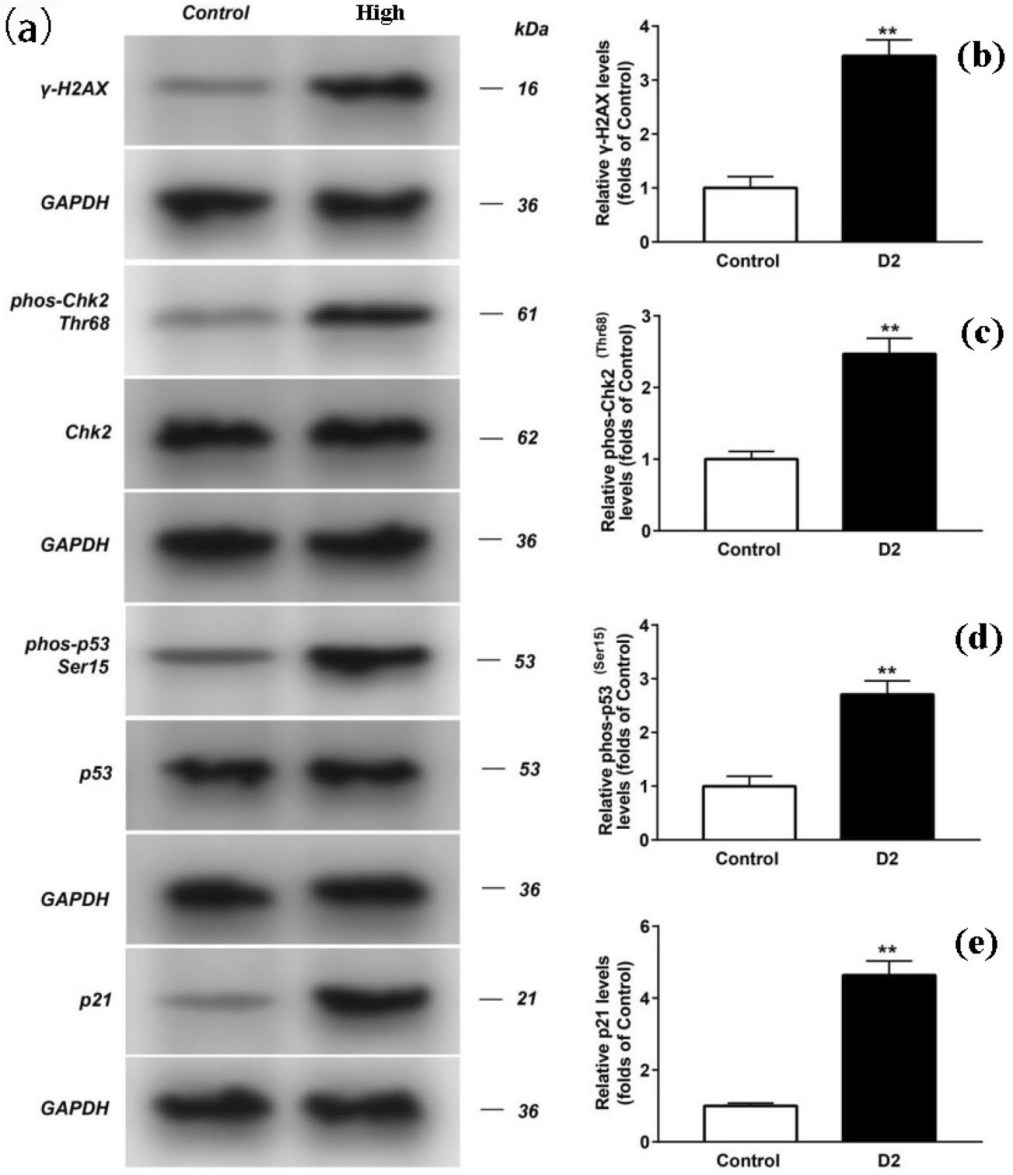
The expression ofγ-H2AX, p21, p53, Chk2, phos-Chk2, and phos-p53 in PC12 from different groups were detected by western blot assay, and representative bands were shown in (**a**). Phos-Chk2 (**c**), phos-p53 (**d**) and the levels of γ-H2AX (**b**), p21 (**e**) were normalized to control (GAPDH). The results were presented as mean ± SD (n = 3). **p < 0.01 vs. Control group. Original full-length gels which are not overexposured were displayed directly. (The originsl high-resolution images of all gels and blots were provided in Supplementary Fig. S12).

Compared with Control group, the content of 8-OHdG was increased significantly. The mRNA expression of H2AX and p21 increased significantly (Fig. 6). The mRNA expression of H2AX (Fig. 7 a) and p21 (Fig. 7b) increased significantly. The phosphorylation level of Chk2 (Fig. 8a and c), p53 (Fig. 8a and d) and the expression level of γ-H2AX (Fig. 8a and b)and p21 (Fig. 8a and e) increased significantly. Original full-length gels which are not overexposured were displayed.

## Discussion

In this study, we compared the transcriptome maps between exposed cells (Low and High group) and unexposed cells (Control group) by RNA-Seq for the ICF experiment of the "SG-III" prototype laser facility. A total of more than 1480 differentially expressed genes (DEGs) were identified by RNA-Seq, which was regarded as an effecutive and sensitive tool to compare the gene expression between cells with different treatments and provide abundant data for further functional analysis subsequently. And then, we preliminarily discussed the DNA damage repair mechanism induced by "SG-III" prototype radiation through ELISA, Westernblot and qRT-PCR experiments.

As a result of analysis of DEGs in Fig. 4, it could be seen that the number of DEGs of the High group is 310 more than that of the Low group, indicating that the radiation dose of High group was intenser, which was more likely to lead to changes in the expression of cellular genes, so as to activate the corresponding signaling pathway and bring about related biochemical information response. We could conclude that the high-energy electromagnetic radiation induced by the the "SG-III" prototype laser facility had a strong penetration and could penetrate glass and walls, resulting in more DEGs in the dose group outside the target range. In particular, 84 dose-dependent genes filtrated from DEGs might play an important role in biological and physiological processes, including 20 positively related genes and 64 negatively related genes among the three groups (Supplementary Table S1 and Supplementary Table S2).

In the analysis of GO function, it could be found that the biological processes related to both Low and High group were "sterol biosynthetic process", "steroid biosynthetic process", "secondary alcohol biosynthetic process", "organic hydroxy compound biosynthetic process", "steroid metabolic process", “lipid biosynthetic process”, “small molecule biosynthetic process”, “inflammatory response”, “cellular response to fibroblast growth factor stimulus”, “regulation of epithelial cell proliferation”, and “negative regulation of fat cell differentiation”. The cellular components related to the Low and High group were “collagen trimer”, “axon terminusl”, “neuron projection terminus”, “distal axon” and “plasma membrane receptor complex”. The molecular functions related to Low and High groups were "cytokine activity", "core promoter sequence-specific DNA binding", "cytokine receptor binding", "heparin binding", "chemokine activity", "receptor regulator activity", "receptor ligand activity", "glycosaminoglycan binding", “DNA binding”, “growth factor binding”, and “neurotrophin binding”, which indicated the treatment with low dose radiation expressed a similar intracellular biological response with and the treatment with high dose radiation in some ways, in accordance with the wayne diagram of DEGs between two groups (Fig. 4).

Among the listed up-regulated genes (Tables 2, 3), the genes which encode Ngfr and Ngf are both evidence of p75 (NTR)-mediated signaling pathway and NGF pathway^7^.The Ngfr is regard as a kind of low affinity receptor which can bind to Ngf, Bdnf, Ntf3 and Ntf4, so it plays an important role in differentiation and survival of specific neuronal populations during development, and it also mediates cell survival as well as death of neural cells^8,9^. The Ngf encodes a secreted protein which is incorporated into a larger complex, and this protein has nerve growth stimulating activity and is involved in the regulation of growth and the differentiation of sympathetic and certain sensory neuron^10^. The Ngf not only participates in the regulation of cell proliferation, differentiation, and neuron myelination, but comes into effect in the in vivo physiological role^11^. The Thrombospondin-1 (Thbs1) is an anti-angiogenic factor and endogenous activator of TGF-β, and the protein encoded by it is related to inflammatory response, epithelial cell proliferation and fibroblast growth factor response. The result revealed that Thbs1 participated in “metabolism of proteins”, “focal adhesion”, “PI3K-Akt signaling pathway” and “MicroRNAs in cancer” (Table 3) at same time. Thbs1 expression was reported to decrease in parallel with that of TGF-β1 and collagen III and it can also activate protein kinase B and decrease apoptotic signaling in suspended fibroblasts^12,13^. The atypical chemokine receptor (Ackr3) encodes a member of the G-protein coupled receptor family and controls chemokine levels and localization via high-affinity chemokine binding. Chemokine binding can lead to beta-arrestin recruitment and activate “MAPK signaling pathway” subsequently. It has been reported that Ackr3 transduced signals via “MEK/ERK pathway” to mediate resistance to apoptosis in glioma cells, and it promoted cell growth and survival. It can also play a regulatory role in heart valve development^14,15^. The protein encoded by the IL1RL1 involved in the inflammatory and immune response is a member of the interleukin 1 receptor family. Studies of the similar gene in mouse revealed that such a receptor could be induced by proinflammatory stimuli and might participate in the function of helper T cells^16,17^.

Among the listed down-regulated genes (Tables 2, 3), Ccl2 mainly regulates development in multiple systems including cardiovascular hematopoietic and nervous systems. And it also plays a role in many diverse cellular functions such as differentiation, tumor growth, metastasis, distribution, activation, effector function, immune surveillance, inflammation response, proliferation and survival in the immune system alone^18^, thus we can observe that Ccl2 was involved in most important GO terms and pathways for this study. Cxcl11 (C-X-C Motif Chemokine Ligand 11) was a potent and physiologic inducer of Cxcr3 internalization after T cell contact with activated endothelial cells as reported and it could also induce calcium release in activated T-cells, thus, it might be necessary in central nervous system diseases concerning T-cell recruitment and immune responses in skin^19^. Studies suggested that Egr1 was a cancer suppressor gene and transcriptional regulator. It mainly regulates the transcription of numerous target genes, and thereby plays an important role in regulating the response to DNA damage, growth factors and ischemia. Egr1 may also play a role in the regulation of cell survival, proliferation and cell death and activate expression of p53/TP53 and TGFB1 to prevent tumor formation. Egr3 is an immediate-early growth response gene induced by mitogenic stimulation, and protein encoded by it participates in the transcriptional regulation of genes in controling biological rhythm and many processes including muscle development, endothelial cell growth, lymphocyte development and neuronal development^20^. Vegfa is one member of the Pdgf/Vegf growth factor family, which can induce endothelial cell proliferation, inhibit apoptosis, promote cell migration, and induces permeabilization of blood vessels^21^. Casp4 encodes a protein that is activator of caspases and plays a essential role in the signaling pathways of necrosis, apoptosis and inflammation^22^. Ntrk2 encodes a member of the NTRK family and this kind of kinase is a membrane-bound receptor and member of the MAPK pathway upon neurotrophin binding. Signaling can lead to cell differentiation through this kinase and control the Ras-PI3 kinase-AKT1 signaling cascade to regulate growth and survival^23^. The Fos encodes leucine zipper proteins that can dimerize with proteins of the Jun family, and it can form the transcription factor complex AP-1, so the Fos proteins have been implicated as regulators of cell proliferation, differentiation, transformation, signal transduction and apoptotic cell death. In sum, we can found that the aboved DEGs significantly enriched in many marked GO terms and pathways (Tables 2, 3) play a pivotal role in important cellular biological and biochemical processes for this ICF experiment, including cell differentiation, proliferation, survival, tumor growth, activation, apoptosis, necrosis, inflammation response, immune surveillance, and effector function.

KEGG analysis of the DEGs showed that the major signaling pathways including “TNF signaling pathway, PI3K-Akt signaling pathway, MAPK signaling pathway, Transcriptional misregulation in cancer, cGMP-PKG signaling pathway, Viral protein interaction with cytokine and cytokine receptor, Focal adhesion, and MicroRNAs in cancer” (Table 3). TNF signaling pathway participates in systemic inflammatory response and is one of the cytokines that make up the acute phase response. TNF can bind two kinds of receptors: TNFR1 (TNF receptor type 1) and TNFR2 (TNF receptor type 2) and trigger activation of many pathways, including NFkB and MAPK pathways. PI3K/AKT signaling pathway can be activated by different types of cellular stimuli and toxins, and can be involved in the regulation of many cellular processes, including cell growth, transcription, translation, cell proliferation, cell movement and glycogen metabolism. Many growth factors can activate members of the PI3K family, which in turn promotes the transformation of one lipid signal molecule PIP2, into another lipid signal molecule PIP3 and such phosphorylated product activates protein kinases into cells as a second messenger to assist in activating Akt, which can regulate key cellular responses in apoptosis, protein synthesis, metabolism and cell cycle by phosphorylating substrates. Abnormalities in PI3K-AKT signaling pathway can cause diseases such as cancer and diabetes. MAPK signal pathway is an important signal transduction system for eukaryotic cells to mediate extracellular signal to intracellular response. The signal related to MAPK pathway is a family of highly conserved protein kinases, and it plays an important role in regulating a variety of physiological processes such as cell growth, proliferation, differentiation, apoptosis and death and participates in signaling transduction after activation of various growth factors, cytokines, mitogen and hormone receptors. Similarity, the pathways of Transcriptional misregulation in cancer and cGMP-PKG signaling pathway also involved in cell proliferation, survival, cycle progression and apoptosis, and Transcriptional misregulation pathway also plays an important role in inhibition of apoptosis and tumor cell growth by regulation of related protein.

Then we use ELISA, Western blot and qRT-PCR experiments to further verify the damage of PC12 cells in terms of DNA and proteins induced by "SG-III" prototype radiation. 8-OHdG can be used as a biomarker of DNA oxidative damage^24^, so the significant expression of 8-OHdG confirmed that the ICF experiment of "SG-III" prototype facility caused serious oxidative damage in DNA. r-H2AX is a biomarker of DNA double-strand break (DSBs), and analysis of -H2AX expression is widely used for DNA damage^25^. DSBs could occur in cells due to many exogenous factors, which are the most prominent causes of DSBs, such as X-rays, and they can be detected by assaying for the presence of theγ-H2AX. Hundreds of H2AX molecules in the chromatin around the break site was phosphorylated at the Serine 139 position within minutes of DSB formation and the formation of γ-H2AX foci attract repair proteins to the DSB site^26^. Thus, the significant expression of 8-OHdG confirmed that ICF experiment of "SG-III" prototype facility lead to DNA double-strand break in cells, thus activating the DNA repair response in cells.

And then, we preliminarily discussed the signaling pathway regarding DNA repair of PC12 cells induced by "SG-III" prototype radiation. The up-regulated expression of γ-H2AX indicates the formation of DNA DSBs (double strand breaks). This damage signal number activates and phosphorylates the downstream protein kinase ATM/ATR (transducers, which can sense DNA damage). Activated ATM/ATR protein kinase continues to transduction DNA damage signals to downstream target proteins p53 (Ser15) and Chk2 (Thr68), which are phosphorylated and activated. Then relay and amplify the DNA damage signal by up-regulating the expression level. In addition, when Chk2 (Thr68) is activated, its substrate p53 protein Ser15 site is phosphorylated. The activation of p53 (Ser15) triggers the transcription of downstream gene p21 and up-regulates CDKs inhibitors, including p21, to participate in the regulation of cell cycle, cause cell cycle stagnation at the G1max S checkpoint. In addition, p53 may affect the G2max M checkpoint through the trans-activation of p21 for DNA repair or apoptosis. In addition, p53 and EGR1 cooperate with each other in controlling cell growth and cell cycle, and the changes of p21 and Gadd45 levels induced by the up-regulation of p53 protein can also lead to G1 and G2 phase arrest of cell cycle. P53 can also induce apoptosis through death signal receptor protein pathway, TNF signal pathway, PI3K-Akt signal pathway, MAPK signal pathway and tumor transcriptional regulation disorder^27–29^.

As the research on electromagnetic pulse radiation of high power laser equipment is in its infancy, there are still many unknown fields to be explored, and we can take some measures to minimize possible occupational risks. First, we can further deepen the understanding of the mechanism of electromagnetic pulse radiation, and find out the law of the generation, propagation and interaction with materials of electromagnetic pulse radiation, to study the methods of reducing electromagnetic pulse radiation from the experimental design. In addition, a special electromagnetic radiation shielding device made of kind of wave-absorbing materia could be designed, because it is light in weight and can be designed as a movable shielding wall to provide effective radiation protection for personnel. We can also strengthen the construction of the remote centralized control system of the shooting range of the high-power laser facility to realize the effective separation between the human and the experimental site and reduce the probability of the personnel being exposed to the radiation environment.

## Summary

In conclusion, we compared the transcriptomes between exposed and unexposed PC12 cells by RNA-Seq. GO analysis of the DEGs suggested that the significantly up-regulated genes Thbs1, Ngfr, Ngf, Fcgr2a, Il1rl1, Ackr3 and the significantly down-regulated genes Egr1, Ccl2, F3, Ccl7, Casp4, Cxcl1, Cxcl11 and Ntrk2. Such DEGs were significantly enriched in the TNF signaling pathway, PI3K-Akt signaling pathway, MAPK signaling pathway, Transcriptional misregulation in cancer, cGMP-PKG signaling pathway, Viral protein interaction with cytokine and cytokine receptor, and MicroRNAs in cancer in KEGG pathways analysis (Table 3). These identified DEGs and pathways were involved in cell growth, proliferation, differentiation, activation, transformation, necrosis, inflammation response, apoptosis and death in vivo during the ICF experiment of the "SG-III" prototype laser facility (Table 2).

Furthermore, we have observed that exposure to such radiation induced up-regulation of 8-OHdG and r-H2AX. The phosphorylation of H2AX indicated the formation of DNA DSBs (double strand breaks), and profoundly activated DNA damage response in cells at the same time. Simultaneous up-regulation of multiple important tumor suppressor and damage repair proteins (phos-Chk2, phos-p21 protein and phos-p53 Ser15) suggests that damage repair mechanisms were activated promptly, but this repair could not reverse the process of DNA damage and finally showed the increase of apoptosis, in accordance with significantly decreased viability of PC12 cells.

## Materials and methods

### Set up

The "SG-III" prototype laser facility shoots high-power pulsed laser beams to illuminate the tiny pellet, which could generate extremely high temperature and extreme pressure instantaneously during the process, and maintain a certain constraint time to achieve ICF reaction to release striking fusion energy. And the profile of facility is shown in Supplementary Fig. S8.

### Main instruments and reagents

CO_2_ incubator was made in Japan from SANYO Electric Biomedical Co., Ltd. Multiskan Spectrum was purchased from American Thermo Fisher Science Co., Ltd. Inverted fluorescence microscope (ICX41) was purchased from Ningbo Shunyu instrument Co., Ltd.

CCK-8 reagent was purchased from APExBIO. Trypsin 0.25% solution, penicillin–streptomycin solution, and DMEM media were purchased from www.gelifesciences.com/hyclone. TRIzol Reagent (Total RNA Isolation Reagent) was purchased from American Thermo Fisher Science Co., Ltd. Fetal bovine serum (FBS) was purchased from Shanghai Zhong Qiao Xin Zhou Biotechnology Co.,Ltd. 24-well, 6-well and 96-well culture plates were purchased from Wuxi NEST Biotechnology Co., Ltd. EP pipes (2, 5, 15 and 50 mL) were purchased from Wuxi NEST Biotechnology Co., Ltd.

### Cell culture and seeding

PC12 cells and HaCat cells were seeded with a suitable density in the proliferation medium (HyClone DMEM) supplemented 10% fetal bovine serum and 1% penicillin–streptomycin and then placed in the cell culture incubator at 37 °C in 5% CO_2_ and 95%-humidified incubators and were sub-cultured three times a week. Appropriate density of cells are seeded into culture plates with area of 109.7 cm^2^ (Supplementary Fig. S9) for experiments when the cells are in the logarithmic growth.

### Irradiation experiment

Since the electromagnetic pulse and the X-ray pulse generated during the process of laser and target coupling have transient and directional instability, the irradiation energy at different positions around the target chamber of the "SG-III" prototype laser facility is different, so radiation dose can be adjusted by placing cells at different locations. In our experiment, cells of High Group was put near the chamber, cells of high-dose group of Low Group was put near the outer door of the shooting range and Control Group was located outside the building of "SG-III" prototype laser facility (Supplementary Fig. S10).

Besides, the detail of radiation flux intensity was measured in precise position of High group, including X-ray (Supplementary Fig. S11).

### Cell proliferation experiment through CCK-8 method

When irradiated cells were incubated for 24 h, the supernatant was discarded and a certain concentration of CCK-8 solution mixed with medium without FBS was added in each well. After incubated for 4–6 h, the cells showed orange color, and the absorbance value (OD value) of each well could be read by the Multiskan Spectrum at the wavelength of 450 nm. The calculation formula of viability: viability (%) = (experimental group OD value—blank control group OD value) / (normal control OD value—blank control group OD value) × 100%.

### Transcriptome sequencing (RNA-Seq) transcriptome based on second-generation sequencing technology

The transcriptome sequencing used in this study is eukaryotic mRNA with reference transcriptome sequencing, which is based on the Illumina sequencing platform. The cDNA sequence generated by mRNA reverse transcription was sequenced by high-throughput sequencing technology, and the RNA information of the sample is obtained, then the molecular mechanism of biological phenomena or disease occurrence is revealed by comparing transcriptome or gene expression profile. The key in RNA-Seq is the significant analysis of gene expression differences. Statistical methods are used to compare the differences of gene expression under two or more conditions, to find out the specific genes related to the conditions, and then to further analyze the biological significance of these specific genes. The analysis process includes six steps: data quality control, reference genome alignment, quantitative gene expression analysis, RNA-Seq correlation analysis, difference significance analysis and functional enrichment. Cell samples saved by TRIzol Reagent sent to Beijing Novogene Bioinformation Technology Co., Ltd. (Beijing, China) for RNA-Seq. Standard methods were used to extract RNA from cell samples, and then carries out strict quality control on RNA samples: NanoPhotometer spectrophotometer is used to detect RNA purity (OD260/280 and OD260/230 ratio) and Agilent 2100 bioanalyzer is used to accurately detection of RNA integrity.

### Library preparation

cDNA library preparation was performed by the Illumina Novaseq 6000 (Novogene Bioinformatics Technology Co., Ltd., Tianjin, China) platform. A total RNA (at least 1 µg) from each sample group (Low, High or Control group) was used for the RNA sample preparations. Sequencing libraries were constructed using the NEBNext UltraTM RNA Library Prep Kit for Illumina (NEB, USA). The mRNA, with polyA tail from total RNA was enriched by poly-T oligo-attached magnetic beads, and then the mRNA was randomly interrupted by divalent cations under elevated temperature in NEBNext First Strand Synthesis Reaction Buffer (5X). First strand cDNA was synthesized using random hexamer primer and M-MuLV Reverse Transcriptase (RNase H). Second strand cDNA synthesis was subsequently performed using DNA Polymerase I and RNase H., and RNA chain was degraded by RNaseH, the second chain of cDNA was synthesized from dNTPs in DNA polymerase I system. After adenylation of 3′ends of DNA fragments, NEBNext Adaptor with hairpin loop structure were ligated to prepare for hybridization. The library fragments were purified with AMPure XP system (Beckman Coulter, Beverly, USA) to select cDNA fragments of preferentially 250–300 bp in length for PCR amplification. Then PCR amplification was conducted and PCR products were purified (AMPure XP system) and library quality was assessed on the Agilent Bioanalyzer 2100 system^30,31^. Finally, the library was obtained. The library products were used for sequencing using Illumina Novaseq 6000.

### Quality control

The image data measured by the high-throughput sequencer are converted into sequence data defined as “raw reads” by CASAVA base recognition. Raw data (raw reads) of fastq format mainly contains the sequence information of the sequencing fragments and their corresponding sequencing quality information. The original data obtained by sequencing contain a small number of reads with sequencing connectors or low sequencing quality. Prior to mapping these reads to the reference database, clean data (clean reads) were obtained by removing reads containing adapter, reads containing ploy-N and low quality reads from raw data. At the same time, Q20, Q30 and GC content the clean data were calculated. All the downstream analyses were based on the clean data with high quality.

### Reads mapping to the reference genome

Reference genome and gene model annotation files could be directly downloaded from genome website directly. Index of the reference genome was built using Hisat2 v2.0.5 and paired-end clean reads were aligned to the reference genome using Hisat2 v2.0.5. Then the mapping tool of Hisat2 was used to generate a database of splice junctions based on the gene model annotation file, so it has a better comparison effect than other non-splicing comparison tools^30^.

### Quantification of gene expression level

FeatureCounts v1.5.0-p3 was used to count the reads numbers mapped to each gene. And then FPKM of each gene was calculated according to the length of the gene and reads count mapped to this gene. FPKM refers to as expected number of Fragments Per Kilobase of transcript sequence per Millions base pairs sequenced and it is the most commonly used method to estimate the level of gene expression, for that it considers the effect of sequencing depth and gene length for the reads count at the same time^32^^.^

Prior to differential gene expression analysis, for each sequenced library, the read counts were adjusted through one scaling normalized factor^33^. Differential expression analysis of two conditions was performed. The *P*-value was adjusted using the Benjamini & Hochberg method. *P*-value of 0.05 and absolute value of “log2(FoldChange) ≥ 1” were set as the threshold for significantly differential expression^34,35^, and sometimes, padj is introduced to correct the *P*-value of the hypothesis test, thereby controlling the proportion of false positives^36^.

### GO and KEGG enrichment analysis of DEGs

Gene Ontology (GO) enrichment analysis of DEGs was realized by the clusterProfiler R package, in which the gene length deviation was corrected. GO terms with corrected *P*-value less than 0.05 were considered significantly enriched by DEGs. KEGG is a database resource for comprehending high-level functions and utilities of the biological system, such as cells, organisms and ecosystems, from molecular level information, especially large-scale molecular data sets generated by genome sequencing and other high-throughput databases. (http://www.genome.jp/kegg/)^37^. We used clusterProfiler software to analyze the statistical enrichment of DDEGs in KEGG pathways.

The first step of gene functional enrichment analysis is to construct gene set, such as GO and KEGG database, that is, genome annotation information for classification. Then map our target gene set (differential gene set or other gene set) to the background gene set and pay attention to distinguish between annotation and enrichment.

Enrichment analysis is based on the principle of hypergeometric distribution, in which the differential gene set is the gene set obtained from the significant difference analysis and annotated to the GO or KEGG database, and the background gene set is the gene set of all the genes analyzed and annotated to the GO or KEGG database. The result of enrichment analysis is to enrich all the differential gene set, up-regulated differential gene set and down-regulated differential gene set of each differential comparison combination. The calculation formula is:

$$ P = 1 = \sum\limits_{m - 1}^{i = 0} {\frac{{\left( {\begin{array}{*{20}{c}} M \\ i \end{array}} \right)\left( {\begin{array}{*{20}{c}} {N - M} \\ {n - i} \end{array}} \right)}}{{\left( {\begin{array}{*{20}{c}} N \\ n \end{array}} \right)}}}  $$

N is the number of all genes annotated to KEGG/GO database, n is the number of DEGs in N, M is the number of all genes annotated to the certain KEGG/GO terms, m is the number of DEGs in M.

### ELISA, western blot and quantitative real-time PCR (qPCR)

ELISA was used to detect the content of 8-hydroxydeoxyguanosine (8-hydmxy-2 deoxyguanine, 8-OHdG) in PC12 cells to verify the oxidative damage of DNA and the content of 8-OHdG in PC12 cells was increased significantly relative to the Control group. The expression of H2AX and p21 (CDKN1A) genes was detected by qPCR method to verify DNA damage and cell cycle arrest. The expression of γ-H2AX, phos-Chk2 (Thr68), Chk2, phos-p53 (Ser15), p53 and p21 (CDKN1A) was detected by Western blot. The originsl high-resolution and full-length images of all gels and blots were provided (Supplementary Fig. S12). mRNA expression levels were normalized by the internal GAPDH control (GAPDH was selected as the internal reference gene), and three independent biological replicates and two technical replicates were conducted. The statistical difference between the two groups was tested by one-way ANOV An and Tukey's test, *P* < 0.05. It was considered that there was a significant difference.

## Supplementary Information

Supplementary Information

## Data Availability

The raw data of transcriptome are available through NovoMagic, which is a data analysis cloud platform independently developed by NovoMagic company. (item number:X101SC19103228-Z01-J002-B2). The analysis of gene differential expression of raw reads is available through small tools provided from NovoMagic cloud platform and some data graphs were conducted by the software of Origin Pro 2018. One-way ANOVA and Tukey’s test were used for statistical differences between test data groups in Western blot, ELISA and Q-PCR experiments. All data generated or analysed during this study are included in this published article (and its Supplementary Information files). The datasets generated during and/or analyzed during the current study are available from the corresponding author on reasonable request.
