# Supplementary material for: Transcriptome profiling of cells exposed to particular and intense electromagnetic radiation emitted by the "SG-III" prototype laser facility
Source: Sci Rep. 2021 Jan 21;11:2017. doi: 10.1038/s41598-021-81642-5 (PMC7820428; doi:10.1038/s41598-021-81642-5)
Supplement: Supplementary file 1 — Supplementary Information [file 41598_2021_81642_MOESM1_ESM.pdf]

Transcriptome profiling of cells exposed to particular and intense electromagnetic radiation emitted by the "SG-III" prototype laser facility

Jiangbin Wei[1], Qiwu Shi[1], Lidan Xiong[2], Guang Xin[3], Tao Yi [4], Yunqing Xiao[4], Wanxia Huang[1*]

1 College of Materials Science and Engineering, Sichuan University, Chengdu 610065,
Sichuan, China. E-mail: shiqiwu@scu.edu.cn; huangwanxia@scu.edu.cn
2 Department of Dermatology, West China Hospital, Sichuan University, Chengdu, 610041, China
3 Laboratory of Ethnopharmacology, West China School of Medicine, West China Hospital, Sichuan University, Chengdu 610041, Sichuan, China
4 Research Center of Laser Fusion, China Academy of Engineering Physics, Mianyang 621900
* huangwanxia@scu.edu.cn

Supplementary Table S1 DEGs of PC12 cells in positive correlation with radiation dose by"SG-Ⅲ" prototype laser facility

GENE_ ID	GENE_ NAME	GENE_DESCRIPTION	Readcount		
			Control	Low	High
**24596**	Ngfr	nerve growth factor receptor	744.1879271	2251.781384	2687.703183
**25420**	Cryab	crystallin%2C alpha B	1186.894528	6642.45554	7689.706756
**25554**	Snai2	snail family transcriptional repressor 2	31.04969223	160.8414509	110.8226999
**84348**	Ackr3	atypical chemokine receptor 3	9.014522926	166.8355453	196.6854359
**116777**	Cdh3	cadherin 3	16.02571291	62.93790876	96.84504516
**192249**	Ehd3	EH-domain containing 3	1292.062386	3469.581566	3683.111878
**287526**	Serpinf1	serpin family F member 1	189.3022778	961.0530547	1034.346314

**290655**	Crlf1	cytokine receptor-like factor 1	147.2351347	523.4841627	601.0390181
**292450**	Raet1e	retinoic acid early transcript 1E%2C transcript variant X2	3.004933005	24.97531065	32.94719487
**298300**	Ttc22	tetratricopeptide repeat domain 22	11.01772	70.93003466	75.87856308
**303730**	Cbx2	chromobox 2	141.2255429	552.4556191	574.0821127
**310738**	Ngf	nerve growth factor	61.0976514	190.8119229	223.6423414
**360467**	Tvp23a	trans-golgi network vesicle protein 23A	3.004933005	25.97432639	30.95038704
**361206**	Slc25a48	solute carrier family 25%2C member 48%2C transcript variant X3	37.05928404	164.8375138	228.6343609
**396527**	Ugt1a2	UDP glucuronosyltransferase 1 family%2C polypeptide A2	1.39E-17	17.98220039	32.94719487
**100912557**	LOC100912557	6.8 kDa mitochondrial proteolipid-like	78.12482832	374.6308182	480.2321454
**102549547**	LOC102549547	-	1.39E-17	13.98613733	16.97273202
**102554838**	LOC102554838	stathmin domain-containing protein 1-like	1.001737281	27.97235788	37.93921446
**NOVEL.167**	-	-	15.02411431	87.91330218	98.84185297
**NOVEL.369**	-	PF00041:Fibronectin type III domain	455.7275173	1337.681987	1586.463674

Supplementary Table S2 DEGs of PC12 cells in negative correlation with radiation dose by"SG-Ⅲ" prototype laser facility

**GENE_ID**	**GENE_NAME**	**GENE_DESCRIPTION**	**Readcount**		
			Control	Low	High
**24330**	Egr1	early growth response 1	4778.627269	267.7361346	114.8163155
**24517**	Junb	JunB proto-oncogene%2C AP-1 transcription factor subunit	3348.344404	712.2981367	665.9352721

**25584**	F3	coagulation factor III%2C tissue factor	2668.258924	524.4831785	461.2624712
**29373**	Bmp2	bone morphogenetic protein 2	455.7275173	132.8690103	114.8163155
**54286**	Prkg1	protein kinase%2C cGMP-dependent%2C type 1	385.6156122	121.8798372	99.84025688
**81503**	Cxcl1	chemokine (C-X-C motif) ligand 1	1166.862555	416.5894791	364.4172922
**83722**	Plk2	polo-like kinase 2	1404.241434	408.5973532	377.396543
**83785**	Vegfa	vascular endothelial growth factor A%2C transcript variant 9	3277.2309	998.0166369	891.574555
**116663**	Dusp6	dual specificity phosphatase 6	6404.221869	1841.185917	1696.288104
**294091**	Ifit2	interferon-induced protein with tetratricopeptide repeats 2	712.1367704	250.7528671	215.6551101
**304005**	Nfkbiz	NFKB inhibitor zeta	859.3717712	138.8631047	99.84025688
**310392**	Slc7a11	solute carrier family 7 member 11	978.56201	162.8394823	121.8051429
**313210**	Abca1	ATP binding cassette subfamily A member 1	1737.773783	284.7194021	249.600843
**314322**	Fos	FBJ osteosarcoma oncogene	1448.311774	111.8896798	46.92484968
**360857**	Rgs16	regulator of G-protein signaling 16	1527.438067	203.7991275	177.7157617
**499497**	Mef2c	myocyte enhancer factor 2C%2C transcript variant X6	3794.055801	1143.872934	1010.38462
**688621**	Tslp	thymic stromal lymphopoietin%2C transcript variant X1	211.337448	64.93594024	40.9344262
**102551819**	LOC102551819	U3 small nucleolar RNA-associated protein 14 homolog B-like%2C transcript variant X2	3290.251682	1039.975298	748.8027963
**103690589**	LOC103690589	uncharacterized LOC103690589	488.7802726	126.8749159	86.86100607
**103690593**	LOC103690593	uncharacterized LOC103690593	486.7770753	55.9447986	19.96794385

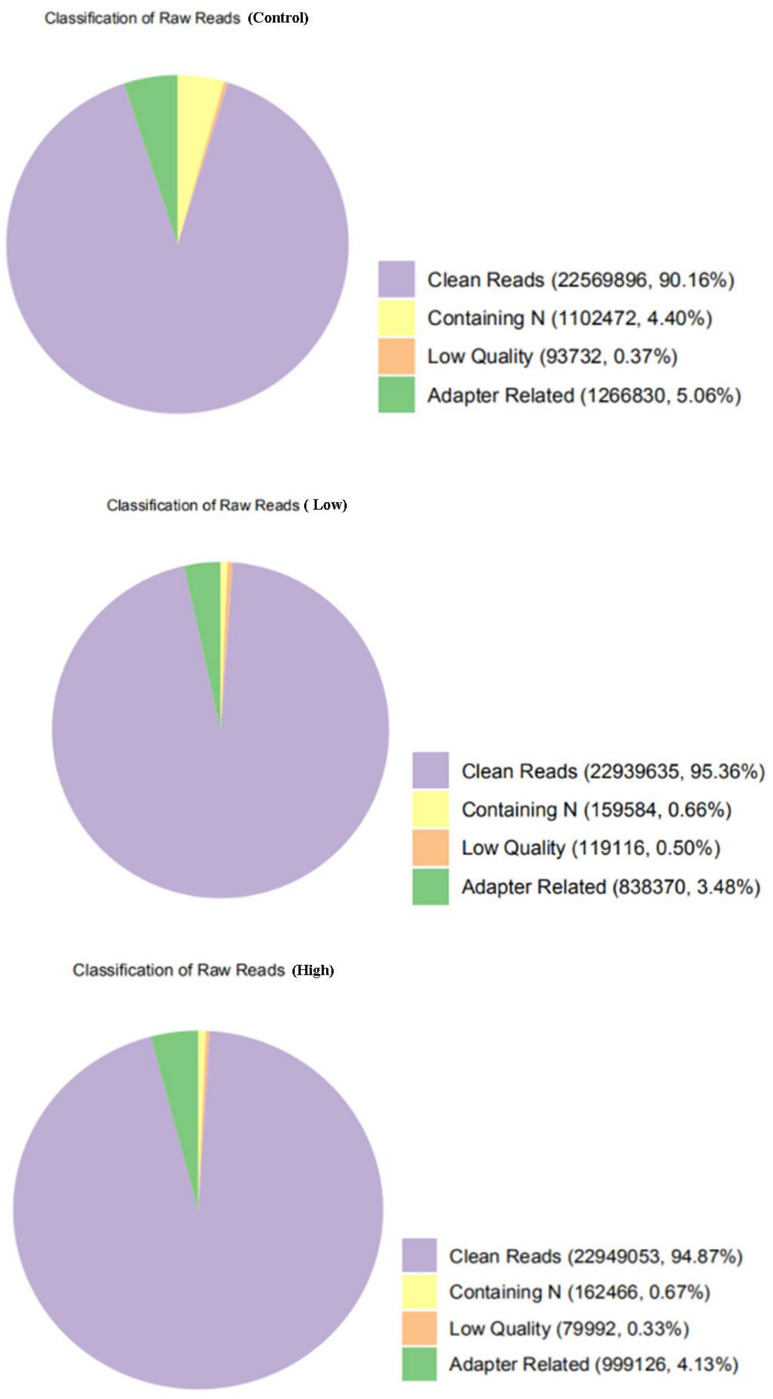

Supplementary Fig. S1 Classification of raw reads of Single-read each group. After filtering reads with containing N, low quality and adapter sequences, the RNA-Seq libraries of CON_B, B2, and D2 generated over 22.5 million clean reads respectively, and the percentage of clean reads among raw reads in each library ranged from 90.16 % to 95.36 %.

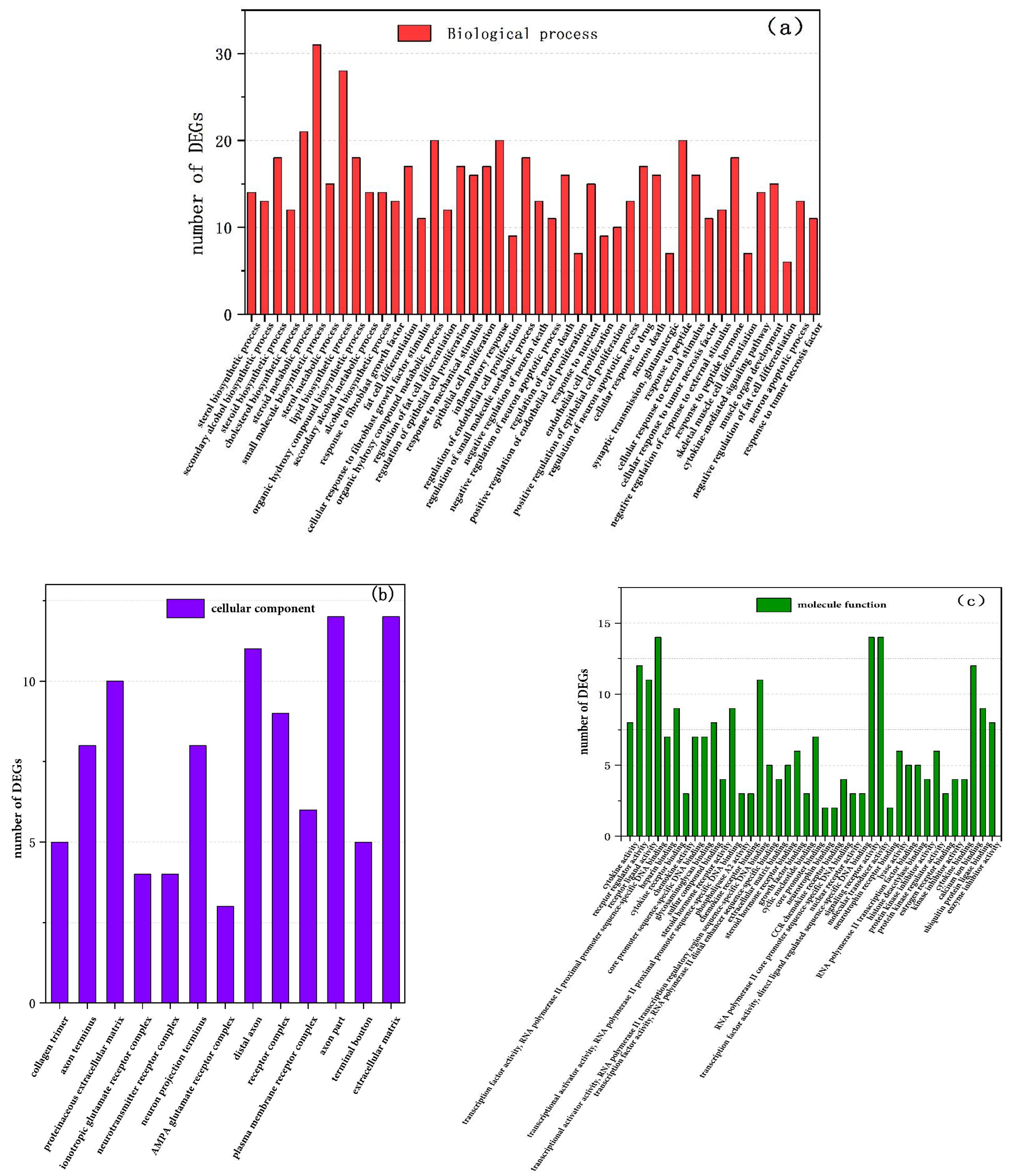

Supplementary Fig. S2 Bar chart of GO enrichment analysis of Low group Vs Control group (a, b, c) and High group Vs Control group (d, e, f). Abscissa in the bar chart is the GO Term, divided into three main domains: biological process (a, d), cellular component (b, e) and molecular function (c, f). In the three main domains, 44, 13 and 40 functional groups were identified respectively in the Low group and 45, 15 and 46 functional groups were identified respectively in the High group . Ordinate is the number of DEGs representing significance level of GO Term enrichment.

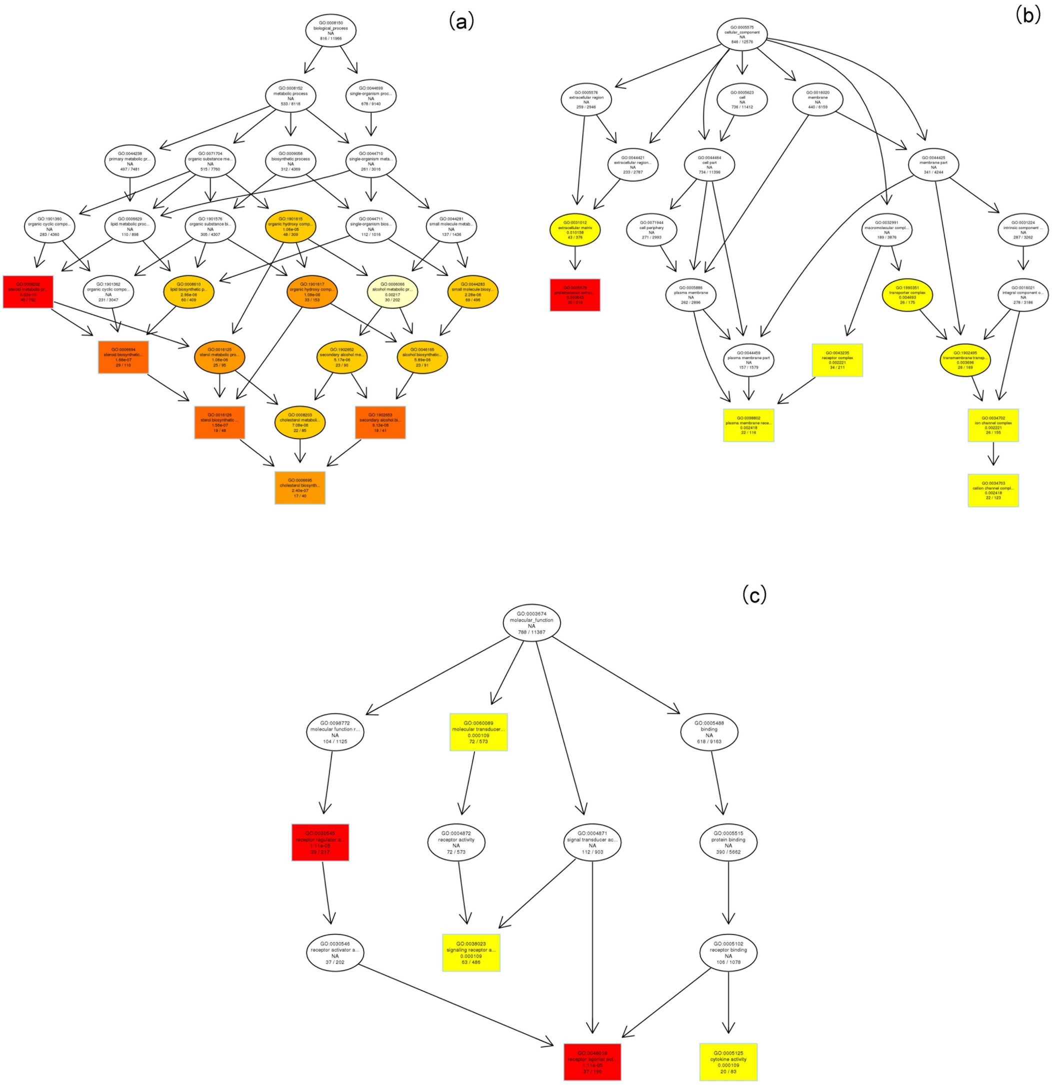
(a)
(b)
(c)
GO:0003674
molecular_function
NA
788 / 11387
GO:0098772
molecular function r...
NA
104 / 1125
GO:0060089
molecular transducer...
0.000109
72 / 573
GO:0005488
binding
NA
618 / 9163
GO:0030545
receptor regulator a...
1.11e-05
39 / 217
GO:0004872
receptor activity
NA
72 / 573
GO:0004871
signal transducer ac...
NA
112 / 903
GO:0005515
protein binding
NA
390 / 5662
GO:0030546
receptor activator a...
NA
37 / 202
GO:0038023
signaling receptor a...
0.000109
63 / 486
GO:0005102
receptor binding
NA
106 / 1078
GO:0048018
receptor agonist act...
1.11e-05
37 / 196
GO:0005125
cytokine activity
0.000109
20 / 83

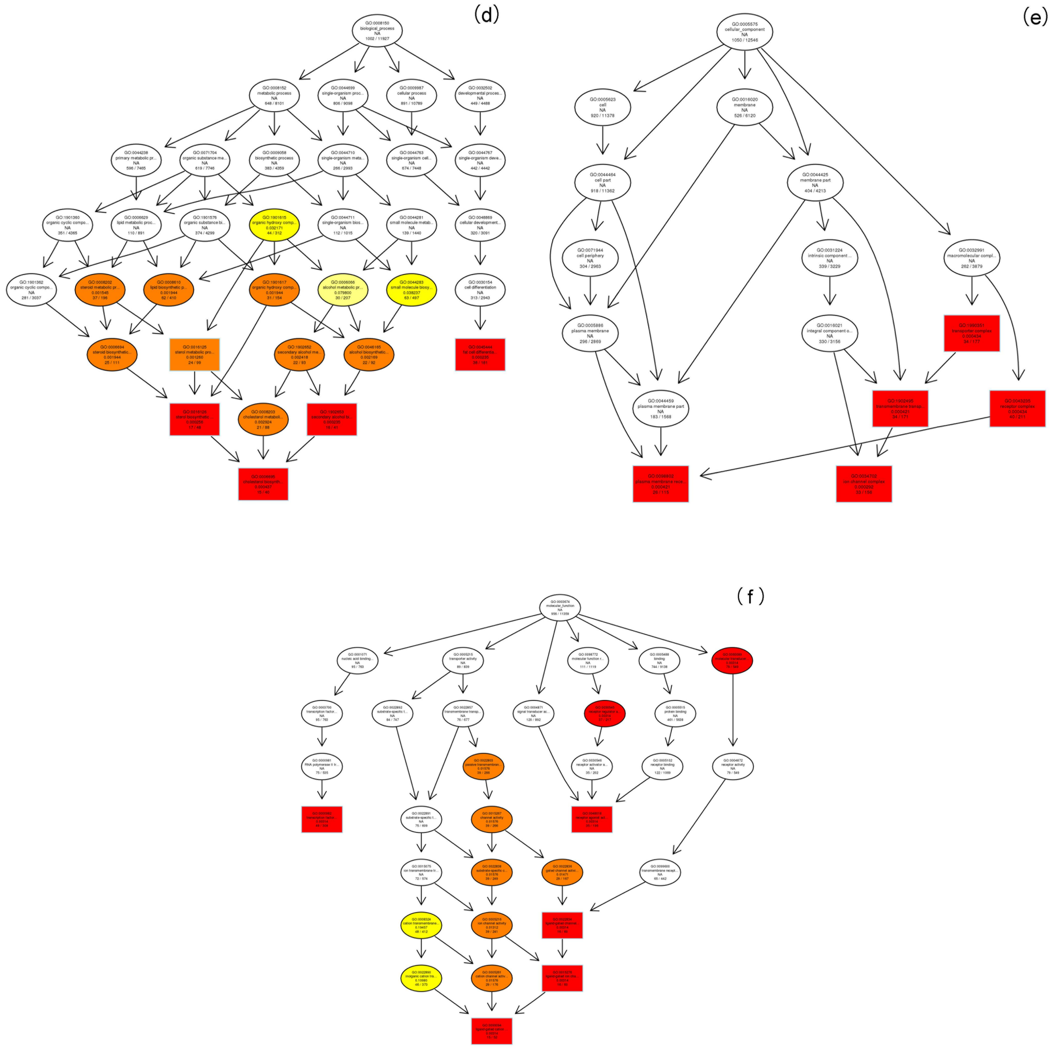

Supplementary Fig. S3 Directed Acyclic Graph (DAG) graph of GO enrichment analysis in Low group Vs Control group (a, b, c) and High group Vs Control group (d, e, f). GO Term is divided into three main domains: biological process (a, d), cellular component (b, e) and molecular function (c, f). Each node represents a GO term. The box represents enrichment degree of TOP5 trems. The darker the color, the higher the enrichment degree. Each node displays The name of the term and the padj of enrichment analysis.

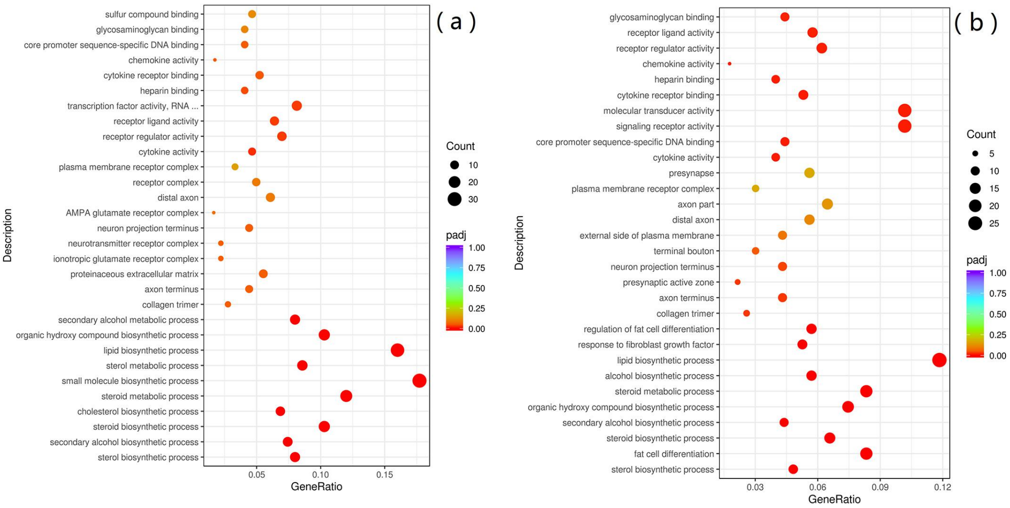

Supplementary Fig. S4 Scatter plot of GO enrichment analysis of Low group Vs Control group (a) and High group Vs Control group (b). (the Abscissa is the ratio of the number of differential genes annotated to GO Term to the total number of differential genes, and the ordinate is GO Term. Database is from www.kegg.jp/kegg/kegg1.html).

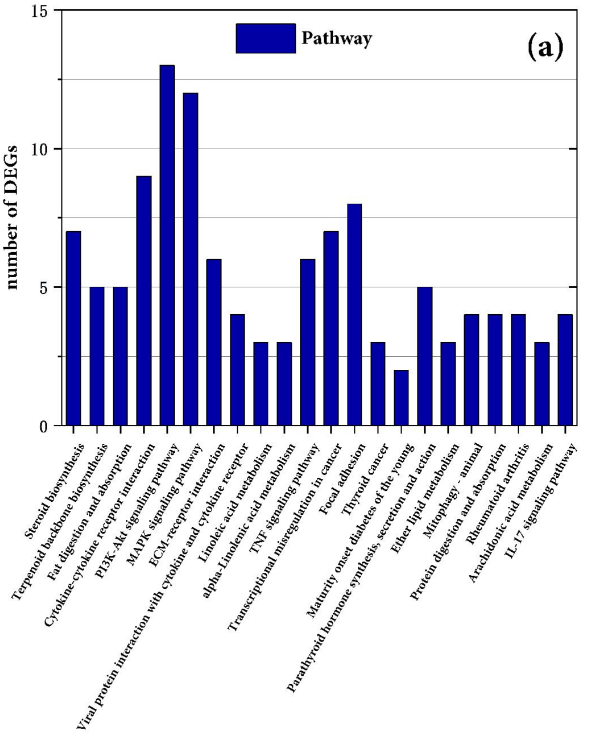

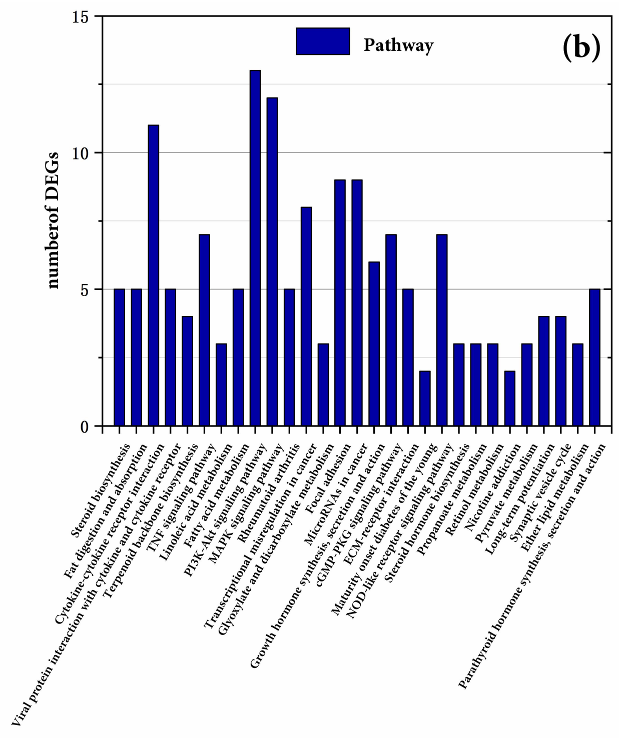

Supplementary Fig. S5 Bar chart of KEGG Pathway analysis of Low group Vs Control group (a) and High group Vs Control group (b). （Abscissa in the bar chart is the pathway, 22 and 29 pathways were identified respectively. Ordinate is the number of DEGs representing significance level of pathways enrichment. Database is from www.kegg.jp/kegg/kegg1.html）

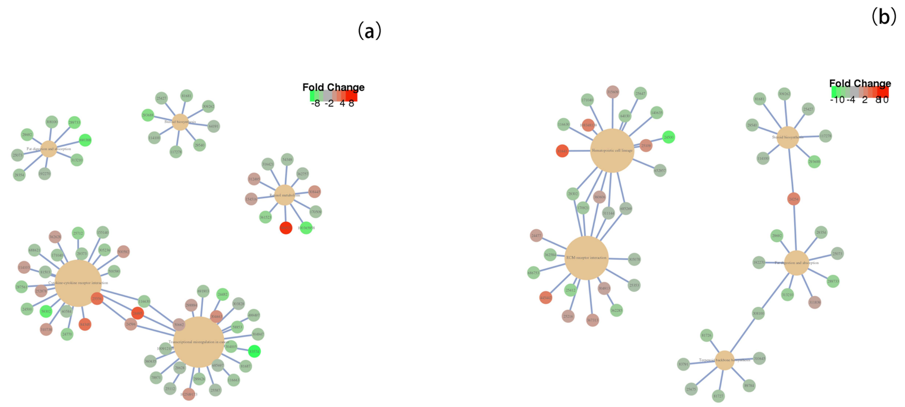

Supplementary Fig. S6 Centpolt graph of pathway analysis in Low group Vs Control group(a) and High group Vs Control group(b). The gray dots in the figure represent genes, and the yellow dots represent enriched pathways. top5 enriched pathways are drawn. The size of the GO node corresponds to the number of enriched DEGs. (Note :Centpolt graph shows the correspondence between genes and enriched GO terms. If a gene is involved in a pathway, they are connected with each other. Database is from www.kegg.jp/kegg/kegg1.html)

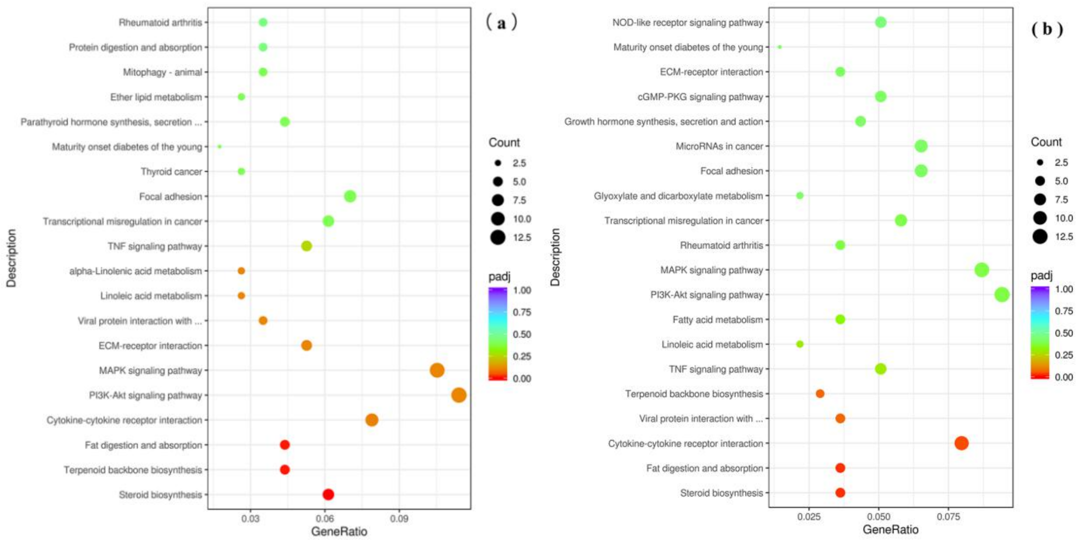

Supplementary Fig. S7 KEGG pathways enrichment and scatter plot of Low group Vs Control (a) and High group Vs Control (b). (The Abscissa is the ratio of the number of differential genes annotated to the KEGG pathway to the total number of differential genes, and the ordinate is the KEGG pathway. The vertical coordinate is the pathway term (with high enrichment degree. Generally, the 20 pathways most significantly enriched in DEGs were identified, Padj is the value range of P-value ranged from [0,1]. The redder it is, the smaller the P-value is, indicating that the enrichment is more obvious. The size of the dot indicates the number of differential genes under the term, and the larger the dot, the more the number of genes. Database is from www.kegg.jp/kegg/kegg1.html)

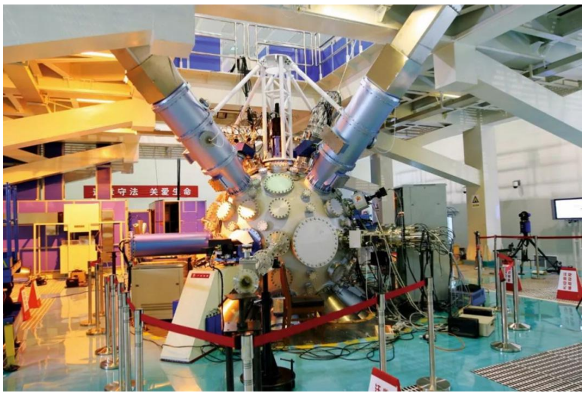

Supplementary Fig. S8 Target range of "SG- III" prototype laser facility [1].

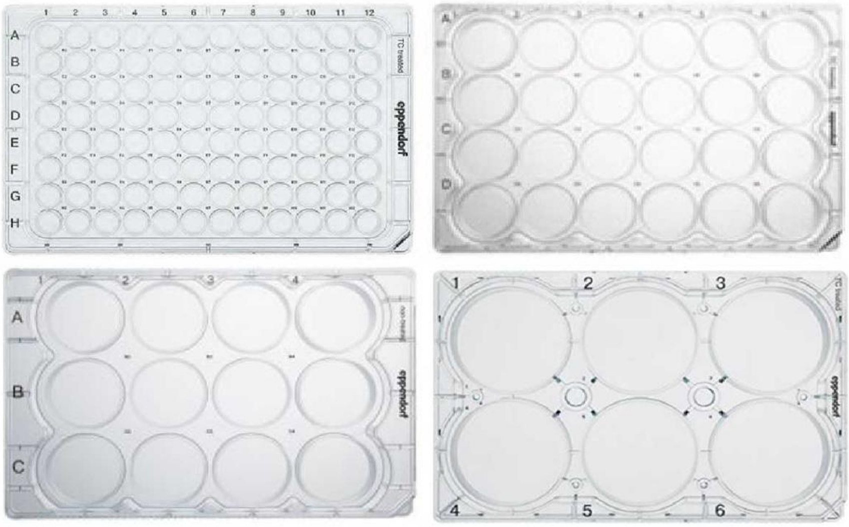

Supplementary Fig. S9 Culture plates with 96-well, 24-well, 12-well and 6-well.

The area is 109.7 cm^2 (127.6 mm * 86 mm)

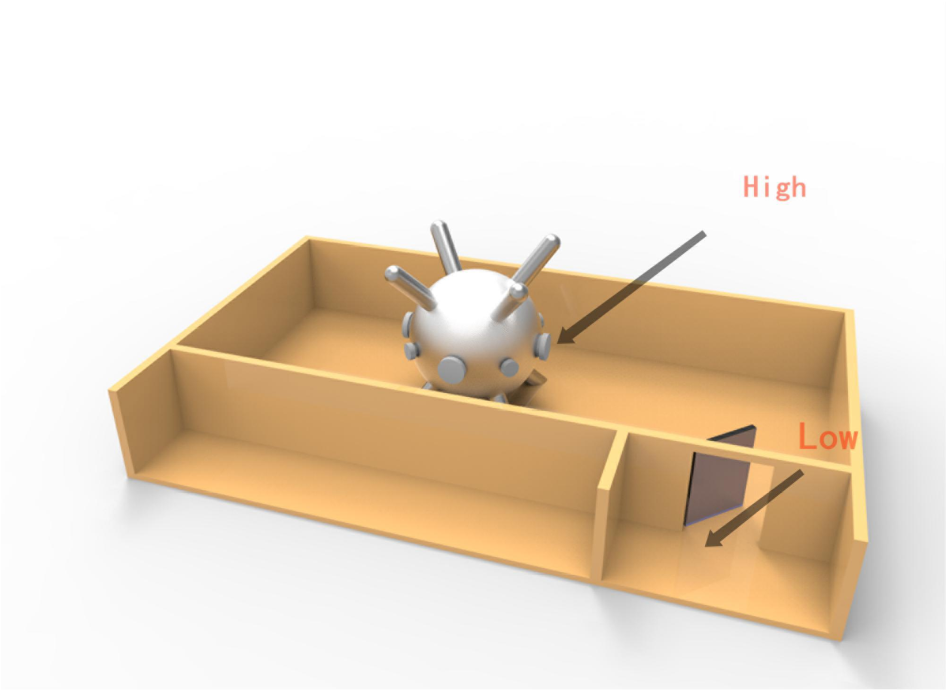

Supplementary Fig. S10 The location of cells of High and Low group.

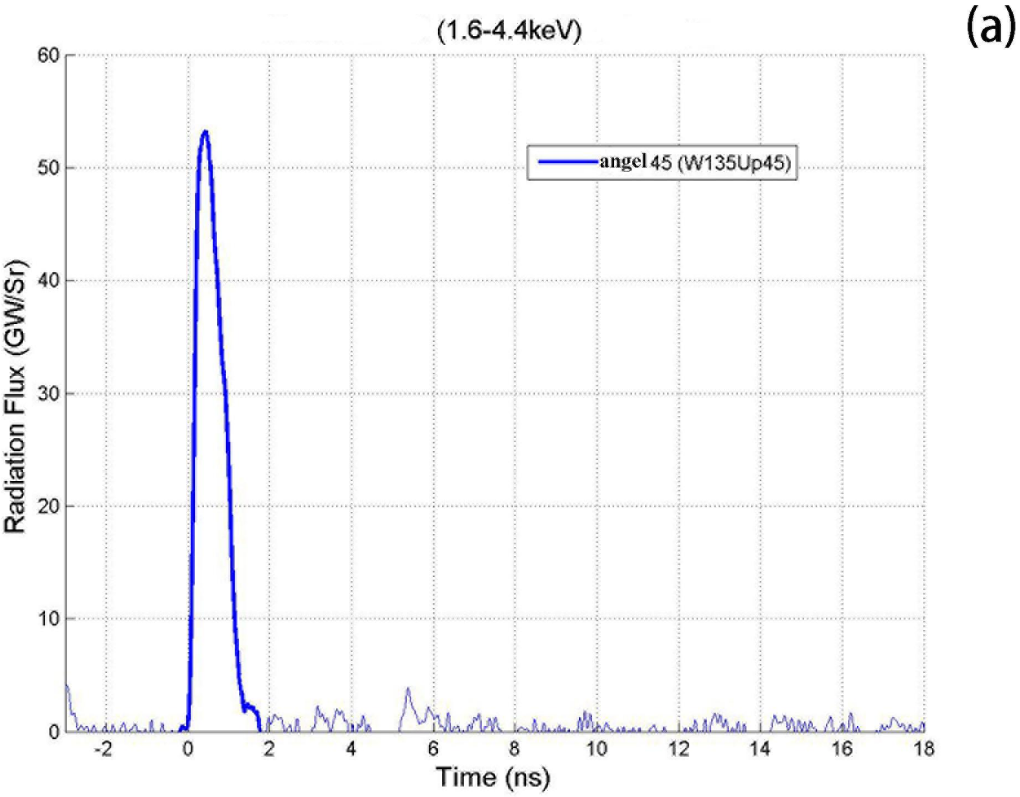

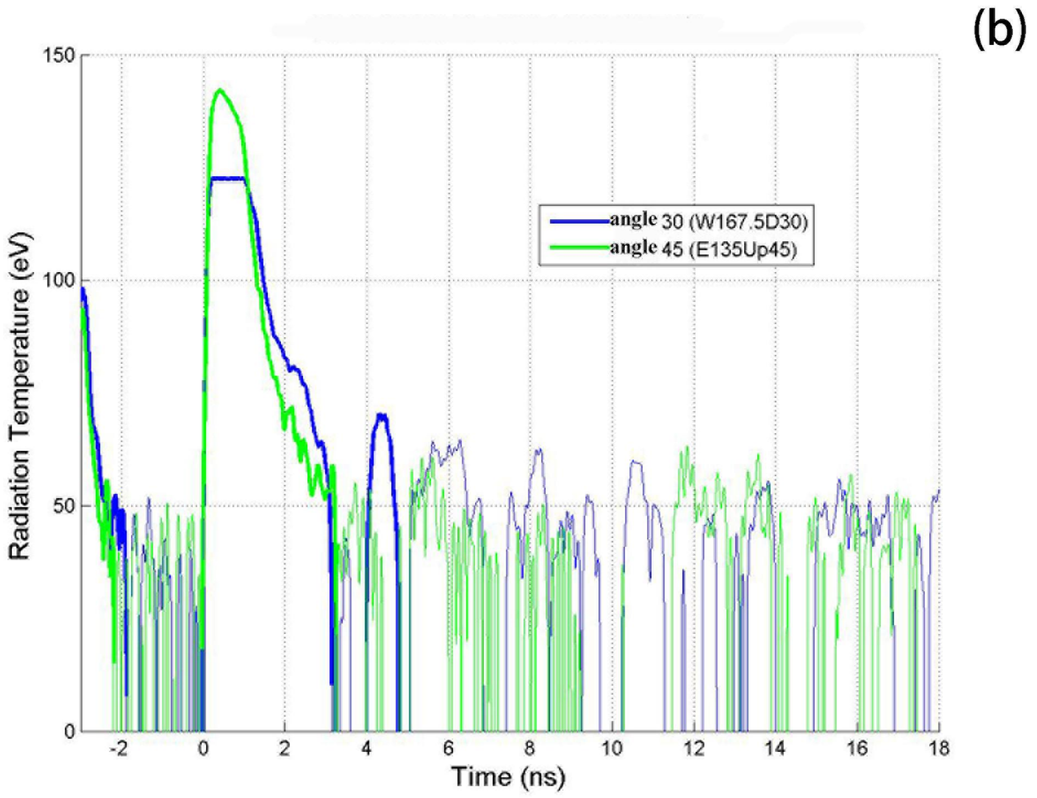

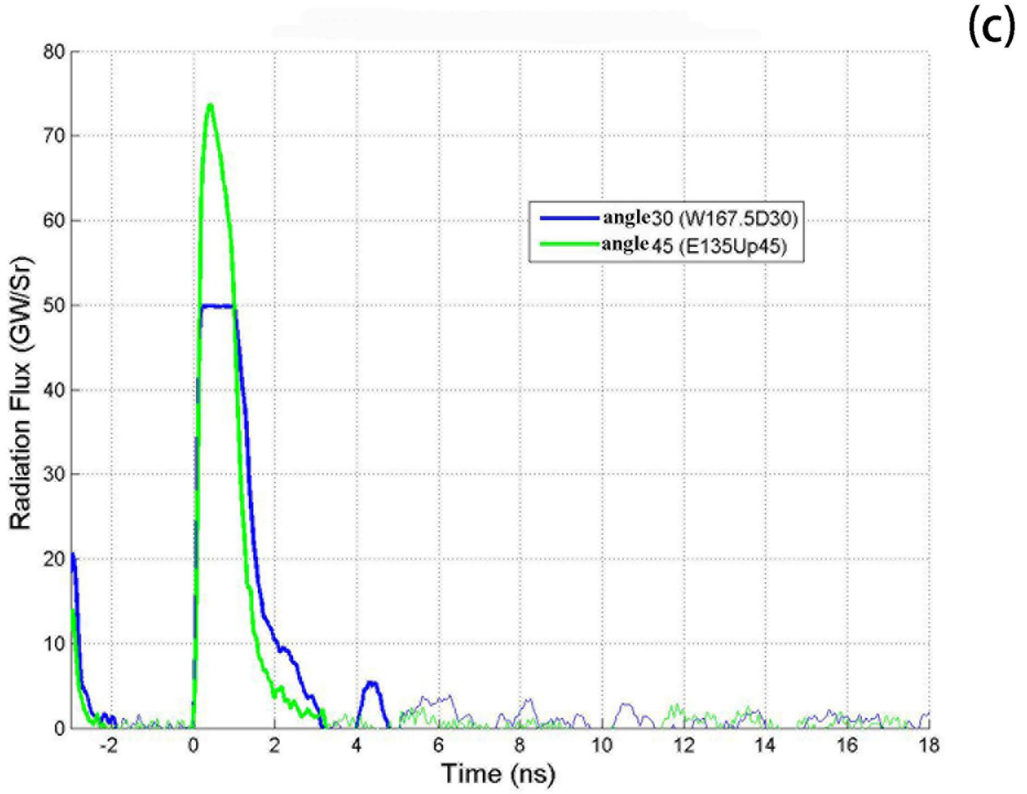

Supplementary Fig. S11 The detail of radiation dose in precise position of High group. Radiation flux intensity(a), radiation temperature(b) and Radiation flux of soft X-ray (c).

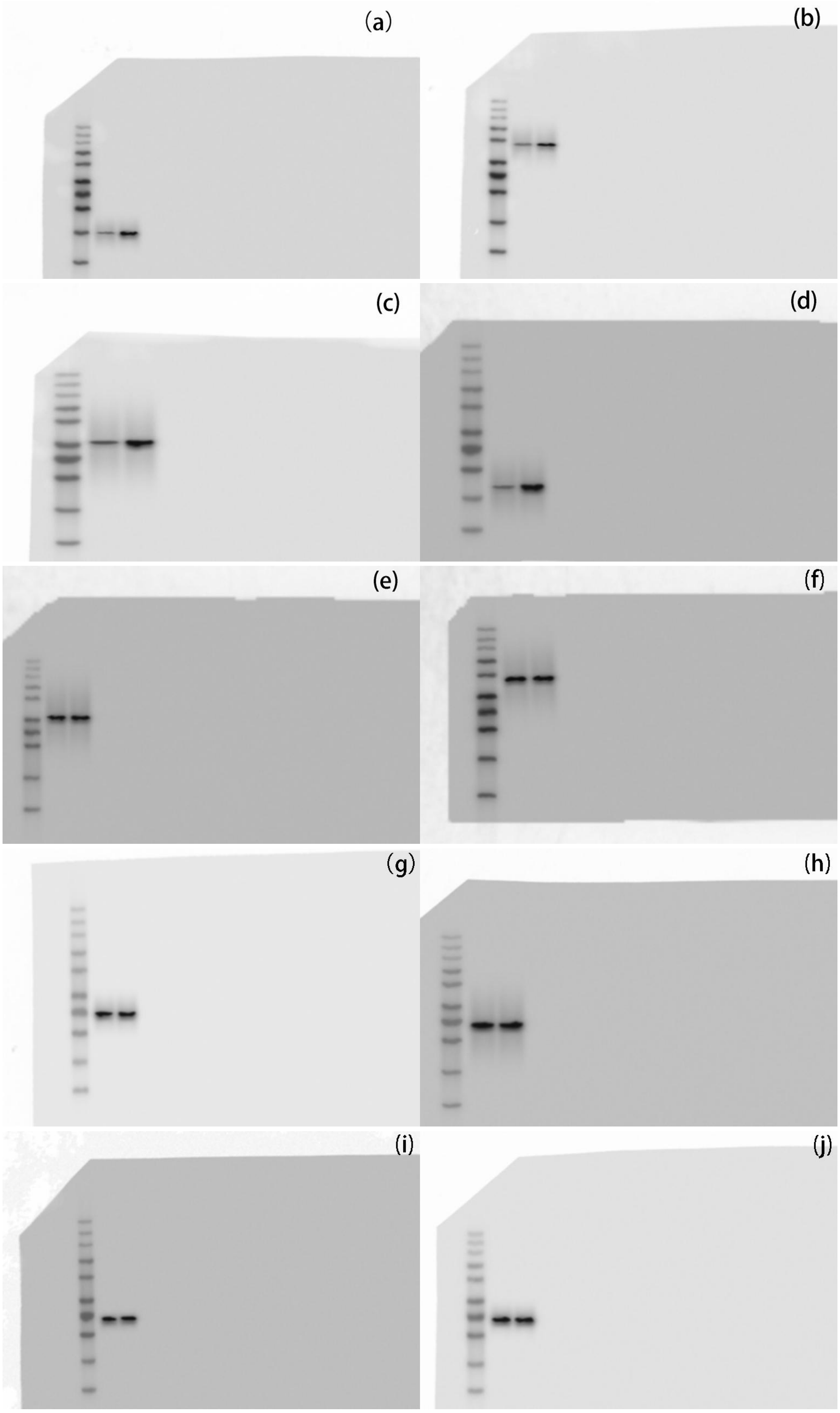

Supplementary Fig. S12 The expression of γ-H2AX (a), p21 (d), p53 (e), Chk2(f), GAPDH (g-j), phos-Chk2 (b), and phos-p53 (c) in PC12 from different groups were detected by western blot assay. All proteins were normalized to control (g-j). The results were presented

as mean ± SD (n = 3). **p < 0.01 vs. Control group. (The originsl high-resolution and full-length images of all gels and blots were provided directly)

References

1. The China National Nuclear Corporation. Read nuclear history daily | Divine SG-III. Available at https://cj.sina.com.cn/articles/view/2369516173/8d3bf28d01900mefw?cre=wappage&mod=r&loc=2&r=9&rfunc=35&tj=none& (2019).
